# Synthesis, Optical Properties and Cellular Toxicity of Water-Soluble near Infrared-II Fluorescent Assemblies Based on Pillar[5]arene

**DOI:** 10.3390/polym15183853

**Published:** 2023-09-21

**Authors:** Qiuxia Wu, Xinran Sun, Zhenming Yang, Pengfei Shi, Huacheng Zhang, Jie Han

**Affiliations:** 1 Frontiers Science Center for New Organic Matter, College of Chemistry, Nankai University, 94 Weijin Road, Tianjin 300071, China; 2 Shandong Provincial Key Laboratory of Detection Technology for Tumor Markers, School of Chemistry and Chemical Engineering, Linyi University, Linyi 276000, China; 3 School of Chemical Engineering and Technology, Xi’an Jiaotong University, Xi’an 710049, China

**Keywords:** NIR-II fluorophore, supramolecular vesicle, water-soluble, cellular toxicity, pillar[5]arene

## Abstract

The main challenges in second near-infrared region molecular fluorophores are poor water solubility and unknown long-term toxicity at present. Herein, new NIR-II molecular fluorophores have been designed and employed to integrate biocompatible pillar[5]arene with 10 outer triethylene oxide groups for the synthesis of rotaxane IRCR. In addition, PEGylated pillar[5]arenes have been combined for the self-assembly of two supramolecular vesicular systems, i.e., PP5-IR1 and PP5-IR2, affording aqueous solubility and lowered cellular toxicity. In aqueous solution, all these fluorophores displayed room-temperature emission with λ_max_ at 986–1013 nm and quantum yields of 0.54–1.45%. They also exhibited good chemical stability and reasonable self-assembled sizes, which may find potential applications in NIR-II imaging. In addition, PP5-IR1 can be used as a fluorescent chemosensor for selective recognition of glutathione through the cleavage of dinitrophenyl ether and release the fluorescent dye.

## 1. Introduction

Based on the excitation and emission wavelengths, the near-infrared (NIR) imaging territories can be mainly categorized into two types: the first near-infrared region (NIR-I, 650–900 nm) and the second near-infrared window (NIR-II, 900–1700 nm) [1,2]. Both of them have better imaging penetration depths in tissues and suffer from less scattering and background fluorescence to achieve high-fidelity visualization in comparison with ultraviolet to visible-light imaging [3]. Compared to the well-established NIR-I fluorescence imaging, NIR-II imaging as a promising method for disease diagnosis and treatment can effectively reduce light scattering, tissue absorption and autofluorescence, leading to the realization of excellent micron-scale resolution, high signal-to-noise ratio and deeper tissue penetration depth [4,5]. Due to the advantages of either bright emission or high photostability and chemical stability, current NIR-II fluorophores have mainly been explored based on inorganic nanomaterials including single-walled carbon nanotubes [6], quantum dots [7] and rare-earth-doped nanoparticles [8]. However, their safety problem has produced limitations in clinical application [9]. In contrast, organic small-molecule dyes (SMDs) with well-defined molecular structure and weight, easy adjustment of optical properties and rapid metabolic ability have shown great potential for NIR-II imaging [10].

Among the SMD NIR-II fluorophores, the small molecules with a donor–acceptor–donor (D-A-D) [11,12,13] or shielding unit–donor–acceptor–donor–shielding unit (S-D-A-D-S) architecture [14] have been widely explored because of their highly tunable electronic structures and good optical properties, and benzobisthiadiazole (BBTD) and its derivatives are commonly used as central electron-accepting aromatic backbones to develop multiple small-molecule dyes [15]. Generally, most of the organic NIR-II fluorophores require further modification of hydrophilic polymer like conjugated connection [16] or physical encapsulation [17] due to low solubility in aqueous solution. Additionally, the size of the SMD complexes and SMD-based organic nanoparticles constructed through such modification could be significantly increased over the renal filtration threshold of ∼40 kDa and inhibit necessary excretion of the fluorescent agent. Furthermore, the large size may result in low fluorescence quantum yields (QYs) because of the enhanced interactions between the conjugated backbone and water molecule [18]. Therefore, substantial improvement in QYs in aqueous solution and coexistence of small size and good solubility in water are the typical issues to address and solve in practical clinical applications.

As a marvelous host molecule, pillar[n]arene has been used widely as part of various water-soluble supramolecular materials in diverse practical fields such as supramolecular gelation systems [19,20,21,22], catalysis [23,24,25] and optical materials [26,27,28,29], as well as adsorption and separation materials [30,31]. Particularly, pillar[n]arene-based amphiphilic supramolecular polymers not only show improved water solubility but also exhibit good performance in terms of the delivery of dyes to cells for bioimaging [32,33]. Although significant research progress in water-soluble pillar[n]arene has been achieved, there has been no report on the integration of pillar[n]arene with NIR-II fluorophores for biomedical applications.

**Scheme 1:**
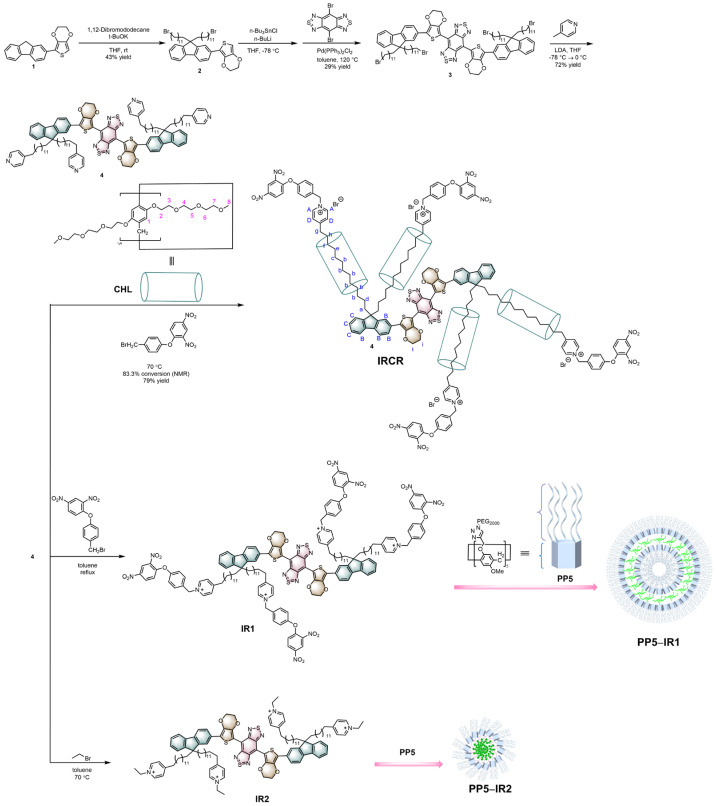
Synthetic route for the compounds **1**–**4** and IRCR, PP5-IR1 and PP5-IR2 (the magenta numbers in CHL and the blue letters in IRCR mean the positions of hydrogens).

Herein, we have designed a small library of pillar[5]arene-based NIR-Ⅱ fluorescent assemblies (IRCR, PP5-IR1, PP5-IR2) (Scheme 1). The NIR-II fluorophore possesses a typical S-D-A-D-S structure with BBTD as the acceptor, 3,4-ethylenedioxy thiophene (EDOT) as the donor and dialkyl fluorene as the shielding unit. The termini of the alkyl chains on fluorene, bearing various pyridinium cations, were used as guest molecules that would be conducive to forming suitable assemblies with pillar[5]arene driven by host–guest interactions and solvophobic effects. Ogoshi et al. described a cyclic host liquid (CHL) as a unique type of solvent, consisting of 10 outer triethylene oxide groups and a pillar[5]arene core, which was biocompatible and miscible with various organic compounds [34]. The CHL system has further proven to be a new powerful approach for high-yield synthesis of mechanically interlocked molecules using the cationization and Huisgen reaction [35]. Thus, CHL was employed here initially for facile and high-yield synthesis of the NIR-II fluorescent rotaxane IRCR. In addition, the tadpole-like amphiphile pillar[5]arene polymers PP5 were also synthesized by introducing five polyethylene glycol (PEG) chains to the pillar[5]arene unit through the “click” reaction. PP5 were used to complex with IR1 and IR2, respectively, to construct the pillar[5]arene-based assemblies PP5-IR1 and PP5-IR2, which presented morphology changes because of size effects. The optical behavior, cellular toxicity and morphology of these fluorophores were investigated. It was found that they all displayed fluorescent emission in the NIR-II region with high QYs in water, low cellular toxicity and reasonable self-assembled sizes. Furthermore, it is noteworthy that PP5-IR1 reacts with glutathione (GSH) smoothly in water, indicating that PP5-IR1 may be used a fluorescent chemosenor for GSH.

## 2. Materials and Methods

### 2.1. General Materials and Methods

Unless otherwise noted, all reagents were obtained commercially and used without further purification. mPEG-N_3_ was purchased directly from Aladdin. Solvents were either employed as purchased or dried according to procedures described in the literature. All air- and moisture-sensitive reactions were carried out in flame-dried glassware under a nitrogen atmosphere. NMR spectra were recorded with a Bruker Avance Ⅲ HD 400 spectrometer (BRUKER, Rheinstetten, Germany). High-resolution mass spectrometry experiments were performed with a Varian 7.0T Fourier transform ion cyclotron resonance mass spectrometer (VARIAN, Palo Alto, CA, USA) or Bruker Daltonics Autoflex III LRF200-CID mass spectrometer (Bruker Daltonics, Billerica, MA, USA). UV-Vis absorption spectra were taken on an Analytikjena specord 210 plus UV-Vis spectrophotometer (Analytik Jenan AG, Jena, Germany). Near infrared (NIR) fluorescence experiments were conducted on an Edinburgh photoluminescence spectrometer (FLS1000) (Edinburgh Instruments, Livingston, Scotland). Fluorescence quantum yields were determined by using the method of Demas and Crosby with quinine sulfate in sulfuric acid (0.1 n) as a standard reference solution (ϕ = 54.6%) [36]. Molecular weights and distributions were determined through size exclusion chromatography (SEC) with a Waters 1525 pump, and THF was used as the eluent. Dynamic light scattering (DLS) measurements were performed in ultra-pure deionized water using a Malvern ZEN1690 (Yip’s Chemical Research and Development Co., Ltd., Shanghai, China). Transmission electron microscopy (TEM) investigations were carried out on an FEI Talos F200X G2 instrument (Thermo Scientific, Needham, MA, USA).

### 2.2. Cell Experiment Materials and Instruments

Phosphate-buffered saline (PBS, pH = 7.4) and a Cell Counting Kit-8 (CCK-8) were purchased from Sangon Biotech Co., Ltd. (Shanghai, China). Dulbecco’s modified Eagle’s medium (DMEM) and penicillin–streptomycin solution were purchased from HyClone (Logan City, UT, USA). Fetal bovine serum (FBS) was purchased from PAN-biotech (Aidenbach, Germany). The human lung adenocarcinoma cancer cell line A549 was purchased from Silver Amethyst Biotech. Co., Ltd. (Beijing, China). All reagents and buffers were autoclaved and used under aseptic conditions. The CCK-8 assay was performed using a microplate reader (EP0CH2, Suzhou Xitogen Biotechnolgies Co., Ltd., Suzhou, China).

### 2.3. Cell Culture

A549 cells were cultured in DMEM supplemented with 10% fetal bovine serum (FBS) and 1% penicillin–streptomycin (10,000 U/mL). The cells were incubated at 37 °C in a humidified atmosphere containing 5% CO_2_. When the cells were grown to about 85%, the medium was discarded and the cells were rinsed with PBS buffer solution once. Then, 1 mL cell lysate was added and the sample was incubated for 1–2 min. The digestion was then terminated with 5 mL DMEM, and cells were collected through centrifugation (800 rpm, 3 min). A cell suspension was prepared by adding 1 mL DMEM and dispersed in medium for cell culture.

### 2.4. Cell Phototoxicity

A549 cells were seeded in a 96-well plate (10^4^ cells per well) and allowed to adhere overnight. The culture medium was replaced with fresh medium containing IRCR, PP5-IR1 or PP5-IR2 at concentrations of 0, 10, 20, 40, 80, 160 and 320 μg/mL. After incubation for 24 or 48 h, 10 μL cck-8 was added to each well, and the absorbance of each well at 450 nm was measured with the microplate reader. The cell viability was evaluated according to the following equation: cell viability (%) = (*A*_test_ − *A*_0_/*A*_control_ − *A*_0_) × 100%. In the formula, “*A*_test_, *A*_0_, and *A*_control_” represent the absorbance of experimental wells with cells and nanoparticles, blank wells without nanoparticles and cells, and control wells with cells but without nanoparticles, respectively.

### 2.5. Methods of Sample Preparation for DLS, Optical Spectroscopy and TEM

Preparation of PP5-IR1: IR1 (2 mg) was dissolved in THF (0.5 mL) and methanol (0.5 mL). Then, the mixture containing IR1 was added into the aqueous solution of PP5 (10 mg) in water (5 mL). After ultrasonic dispersion, the resulting mixture was sealed in dialysis bags with a molecular weight cut-off of 2 kDa to remove small molecular organic solvents.

Preparation of PP5-IR2: IR2 (2 mg) was directly added to a solution of PP5 (10 mg) in water (5 mL) and dissolved through ultrasonic dispersion at 30 °C.

The prepared PP5-IR1 and PP5-IR2 were used directly for TEM measurements. First, 40 μL of the prepared PP5-IR1 or PP5-IR2 was diluted with ultra-pure deionized water (8 mL) and was used for the DLS measurements. The solutions with different concentrations of IR1, IR2, IRCR, PP5-IR1 and PP5-IR2 were prepared using the common method for optical spectroscopy.

## 3. Results and Discussion

Pyridinium derivatives have been fully demonstrated to facilitate the construction of stable host–guest complexes with pillar[5]arenes because of cationic—π interactions [37]. The cationization between the pyridyl end of pseudorotaxane that was composed of the axle **4** (Scheme S1) and the wheel CHL, as well as the stopper 1-(4-(bromomethyl) phenoxy)-2,4-dinitrobenzene, afforded rotaxane IRCR in a high yield (79%) (Scheme 1). All intermediate compounds and the final product were characterized by means of solution proton nuclear magnetic resonance (^1^H NMR), carbon-13 nuclear magnetic resonance (^13^C NMR) and high-resolution mass spectroscopy (HRMS) (Figures S1–S18). In the molecular composition of 1 mol of IRCR, there is about 3 mol of CHL passing through the alkyl chains on fluorene, detected by comparing the integral between H_i_ (methylene protons from EDOT) and H_1_ (phenyl protons of CHL) (Figure S10), which is consistent with the results shown by matrix-assisted laser desorption ionization–time-of-flight mass spectrometry (MALDI-TOF MS) (Figure S11). The calculated M*_w_* difference in **4**/IRCR (1841/8923) indicated approximately three CHLs threaded into each fluorophore molecule. It is noteworthy that the molecular mass of 8.9 kDa for IRCR was well within the size limit of about 40 kDa for renal excretion [18].

**Figure 1:**
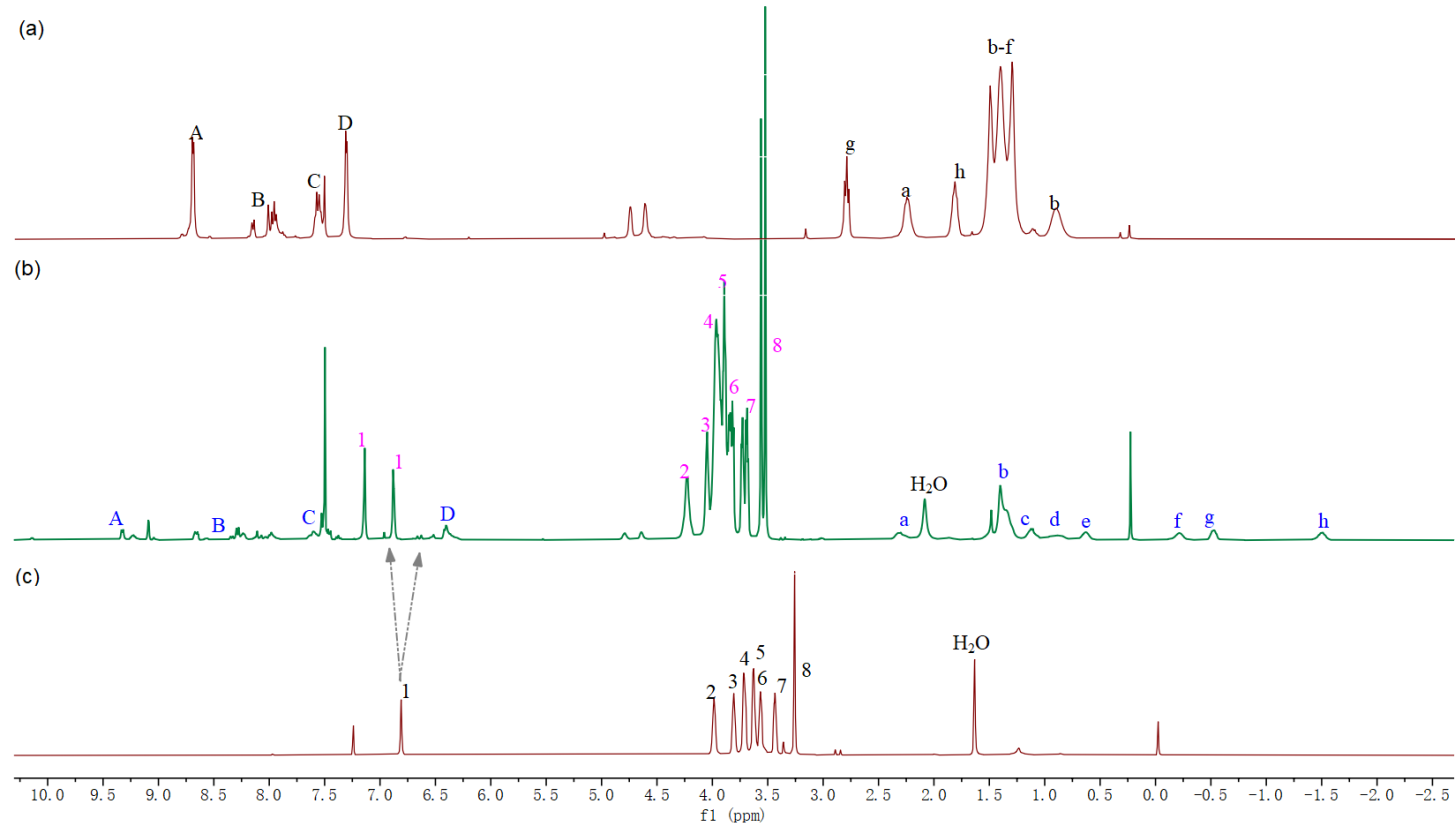
^1^HNMR spectra of (CDCl_3_, 400 M, 298 K) (**a**) **4**, (**b**) IRCR and (**c**) CHL (the molecular structures of **4**, IRCR and CHL with the labels are shown in Scheme 1).

The ^1^H NMR spectra (Figure 1) of the rotaxane IRCR, the axle **4** and the wheel CHL were compared to determine the location of the wheel segment and attribution of protons in rotaxane. Downfield shifts of the H_A_ proton signals were evidence of the successful conversion of pyridine to pyridinium salts. Signals from the phenyl protons H_1_ of the wheel showed downfield shifts because of the deshielding caused by the axle. Upfield shifts of the H_b_-H_h_ proton signals from alkyl chains and H_D_ proton signals from pyridinium salts were observed owing to the aromatic shielding that resulted from the wheel. The results indicated the completion of the assembly of the pillar[5]arene CHL with the long alkyl chains. Meanwhile, the COSY and NOESY studies (Figures S12 and S13) showed two main correlations: one is the correlations between the signals from the phenyl protons H_1_ of the wheel and methylene protons H_e_, H_f_, H_g_ and H_h_ of the axle; the other is the correlations between the methylene protons H_2_ adjacent to the *O*-atom of the wheel and methylene protons H_c_, H_f_ and H_g_ of the axle. This provided further evidence for the formation of rotaxane IRCR. The fact that the pyridinium salts were located at the end closer to the electron-rich cavity of the pillar[5]arene was also consistent with our speculation.

**Figure 2:**
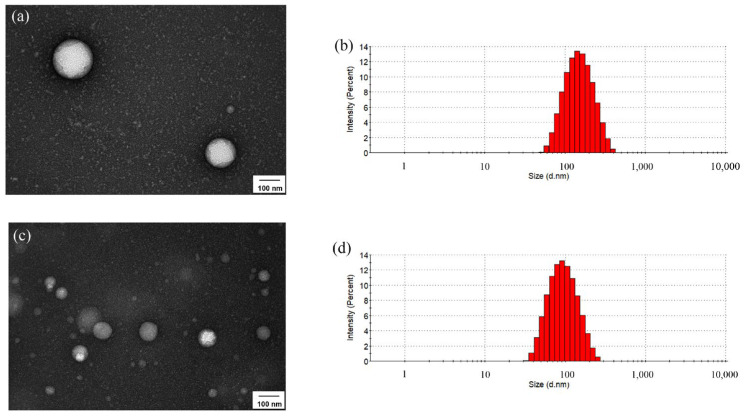
TEM images of (**a**) PP5-IR1 and (**c**) PP5-IR2. Size distribution of (**b**) PP5-IR1 and (**d**) PP5-IR2.

After the establishment of the efficient host–guest recognition based on the pillar[5]arene CHL and the pyridinium salt, the self-assembly behaviors of the amphiphilic supramolecular polymer PP5 with the PEG segment as the hydrophilic part and two bulky NIR-II fluorescent molecules IR1 and IR2 as the hydrophobic section in water were further investigated, respectively. There was no Tyndall effect for the aqueous solution of PP5 (Figure S19a). In contrast, upon the addition of IR1, the nearly transparent aqueous solution of PP5 showed a significant Tyndall effect (Figure S19b), indicating the existence of abundant nanoparticles, whereas the complexation of PP5 and IR2 demonstrated a weaker Tyndall effect (Figure S19c). Then, transmission electron microscopy (TEM) and dynamic light scattering (DLS) measurements (Figure 2) were taken to determine the morphology and size of these nanoaggregates. Negatively stained TEM images clearly showed spherical aggregates, with PP5-IR2 exhibiting a smaller size of 80 nm in diameter and PP5-IR1 exhibiting a larger particle size of 150 nm, which was in good agreement with the DLS results. The spherical aggregates visualized in the TEM images do not have a crystal lattice as they are not crystalline materials, while the black area is the substrate covered with uranium tetraacetate as dye and fixative, so there is an apparent contrast between the particles and the substrate. It was proposed that IR1, with a larger conjugated backbone and pyridinium moieties at the end of the alkyl chain than the cavity of pillar[5]arene, was included by hydrophobic layers of vesicular structures formed by tadpole-like amphiphilic pillararenes. Nevertheless, due to the “lock and key” theory and the matching molecular size, the pyridinium salts of IR2 could thread into the cavity of PP5 without being encapsulated, which greatly increased the biocompatibility of IR2 with bulky hydrophobic size, thus explaining the phenomenon regarding its weaker Tyndall effect and size nearly half that of PP5-IR1. All these results suggested that PP5-IR2 had lower aggregation and better dispersity than PP5-IR1.

The *para*-dinitrophenoxy benzyl pyridinium unit could be used to construct a fluorescent chemosensor for selective recognition of glutathione (GSH) through the cleavage of dinitrophenyl ether and release the fluorescent dye [38]. We attempted to explore the possibilities of IRCR and PP5-IR1 assemblies for recognition of GSH, because both of them contained such pyridinium moieties. The reaction of IR1 and GSH was initially investigated, and identifications of the resulting products were carried out through ^1^H NMR and ESI-MS measurements. The CDCl_3_ solution of IR1 could be sustained for a week as there were no changes in its ^1^H NMR spectrum (Figure S20a,b). After adding GSH to the solution, the methylene protons adjacent to the nitrogen atom of the pyridine salts gradually disappeared (Figure S20c), and the ^1^H NMR spectrum after 7 days of placement showed their complete disappearance along with the appearance of a new peak in the nearby high field, which was direct evidence of the breakage of the C-N bond (as shown in the blue dashed box of Figure S20d). The ESI-MS spectrum (Figure S21) suggested the resultant chemical structure of the fluorophore was identical to that of compound **4**, demonstrating that the expected recognition and self-immolative cleavage had occurred accordingly. The reaction between PP5-IR1 and GSH was investigated under the same conditions as those of IR1 and GSH. As expected, both the attenuated Tyndall effect (Figure S19d) and the reduced sizes of PP5-IR1 (Figure S22) indicated the smooth reaction of PP5-IR1 with GSH.

However, the ^1^H NMR spectra of IRCR upon addition of GSH showed only a simple superposition of IRCR and GSH, and this was not accompanied by the presence of new peaks and the disappearance of old ones (Figure S23). The corresponding NOESY spectra also provided evidence for the continued presence of the long alkyl chains’ correlation with the phenyl protons H_1_ from CHL (Figures S24 and S25). The reason for the lack of dissociation of rotaxane was speculated to be that the TEO chains of CHL tightly wrapped around the pyridinium moieties, causing difficulties for the attacking of the small molecular weight and volume of GSH towards the stopper.

The UV–vis absorption and photoluminescence properties in methanol and aqueous solution for IR1, IR2 and IRCR are collected in Table 1. The maximum absorption peaks of these three fluorophores in methanol solution were all around 762 nm. In contrast, the absorption spectra of PP5 modified fluorophores in aqueous solution were substantially red-shifted, with the peak absorption located at 782 and 796 nm for PP5-IR1 and PP5-IR2 respectively. (Figure S26a). These red shifts could be attributed to the larger polarity of water than that of CH_3_OH, which increases solvent relaxation and reduces the gap between the ground state and excited state. Additionally, the introduced bulky PP5 can limit the rotation of the conjugated backbone.

Unlike the absorption spectra, the emissions of PP5-IR1, PP5-IR2 and IRCR in aqueous solution maintained a high degree of similarity to those in CH_3_OH solution. PP5-IR1 and PP5-IR2 exhibited similar fluorescent emission spectra under 808 nm excitation, with the peak at around 1013 nm and tails extending the emission into the NIR-II region (1300–1400 nm), while there was a blue shift for IRCR with the peak at 989 nm (Figure S26b). Notably, the Stokes shift for all of the samples was larger than 216 nm, suggesting that the overlap between the absorption and emission was very little. The fluorescence QY of IRCR was 0.54% in CH_3_OH, substantially higher than those of IR1 (0.24%) and IR2 (0.24%), suggesting that the protection of water-soluble pillar[5]arene could enhance the fluorescence QY. We further measured the fluorescence QY in water and observed a similar trend: the QY of IRCR (1.45%) was also significantly higher than those of PP5-IR1 (0.61%) and PP5-IR2 (0.54%). Compared to CH_3_OH solution, the further assembly of the amphiphilic pillar[5]arene in water may be the reason for reducing the interaction between water molecules and fluorophores and thus improving the quantum yield.

The fluorescence intensity of IRCR, PP5-IR1 and PP5-IR2 in water did not decay evidently even after the solutions were placed in air for 7 days (Figure S27), indicating that they all showed good chemical stability in water. Meanwhile, the co-existence of IRCR and various analytes such as common amino acids and anions, even in strongly acidic environments, also had no obvious impact on the fluorescence intensity, reflecting excellent anti-interference performance (Figure S28).

**Figure 3:**
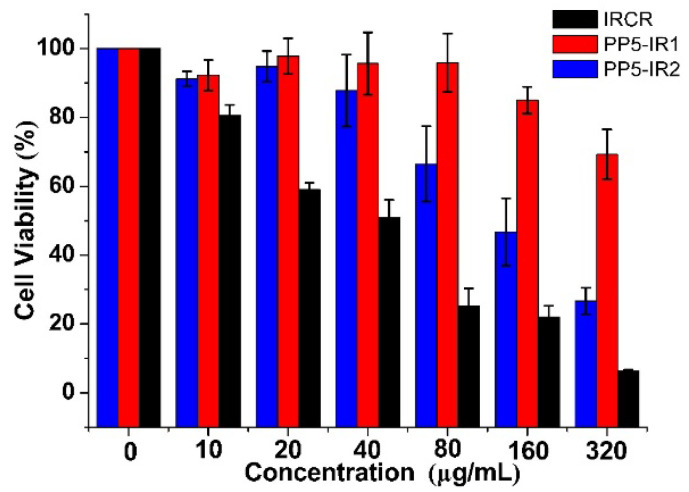
The CCK-8 experimental results of A549 cells incubated with IRCR, PP5-IR1 or PP5-IR2 for 24 h.

The cell counting kit-8 (CCK-8) experimental results of A549 cells incubated with IRCR, PP5-IR1 or PP5-IR2 for 24 h are shown in Figure 3. The survival rate of the A549 cells remained above 80% when the PP5-IR1 concentration was lower than 160 μg/mL. The survival rate of the A549 cells was above 80% when the PP5-IR2 concentration was lower than 40 μg/mL. Compared to PP5-IR1 and PP5-IR2, IRCR showed more obvious cytotoxicity. In addition, IRCR and PP5-IR2 showed a dose-dependent cytotoxicity to A549 cells, that is, the survival rate of the A549 cells decreased with the increase in the corresponding material concentration. As the incubation time was increased to 48 h (Figure S29), the toxicity of PP5-IR1 to the A549 cells was significantly enhanced. When the concentration of PP5-IR1 was lower than 20 μg/mL, the survival rate of the A549 cells was above 80%. In contrast, when the concentration of PP5-IR2 was lower than 40 μg/mL, the survival rate of the A549 cells was more than 80%, indicating that PP5-IR2 has a much lower toxicity than PP5-IR1. This is consistent with the result of 24 h incubation. Overall, IRCR, PP5-IR1 and PP5-IR2 were less cytotoxic. However, relatively speaking, the improved aqueous solubility lowered the cytotoxicity of the obtained nanoparticles PP5-IR1 and PP5-IR2, while IRCR had to be dissolved in water containing a small amount of EtOH (5% volume ratio), with slightly high toxicity.

## 4. Conclusions

In summary, we successfully developed a family of NIR-II molecular fluorophores IRCR, PP5-IR1 and PP5-IR2 based on the assembly of pillar[5]arene with improved fluorescence characteristics in aqueous solutions and different morphological sizes. All these fluorophores in aqueous solution displayed room-temperature emission with λ_max_ at 986–1013 nm and quantum yields of 0.54–1.45%. Rotaxane IRCR, with the highest quantum yield of 1.45%, exhibited a molecular mass of 8.9 kDa, well within the size limit of about 40 kDa for renal excretion. PP5-IR1, with a hydrodynamic size of 150 nm, could efficiently recognize glutathione, thereby reducing the morphological size, while PP5-IR2, which was only half the size of PP5-IR1, had the highest water solubility among the three NIR-II molecular fluorophores. The good chemical stability, high fluorescent quantum yield in water, low cellular toxicity and optimal molecular size of the novel pillararene-based fluorophores may find potential applications in NIR-II imaging.

## Figures and Tables

**Table 1:** The optical properties of the solution of the synthesized fluorophores at 298 K.

Solvent	Sample	λ_abs_ (nm) (logε)	λ_em_ (nm)	Stokes Shift (nm)	QY (%)
MeOH	IR1	762 (3.98)	1016	254	0.24
MeOH	IR2	761 (3.99)	1013	252	0.24
MeOH	IRCR	762 (4.09)	989	227	0.54
H_2_O	PP5-IR1	782 (3.89)	1007	225	0.61
H_2_O	PP5-IR2	796 (3.92)	1013	217	0.54
H_2_O	IRCR	770 (4.04)	986	216	1.45

## Data Availability

The data presented in this study are available on request from the corresponding author.

## Supplementary Materials

The following supporting information can be downloaded at: https://www.mdpi.com/article/10.3390/polym15183853/s1, Figure S1: ^1^H NMR spectrum (400 MHz, chloroform-*d*, 298 K) of **2**; Figure S2: ^13^C NMR spectrum (101 MHz, chloroform-*d*, 298 K) of **2**; Figure S3: Electrospray ionization mass spectrum of **2**; Figure S4: ^1^H NMR spectrum (400 MHz, chloroform-*d*, 298 K) of **3**; Figure S5: ^13^C NMR spectrum (101 MHz, chloroform-*d*, 298 K) of **3**; Figure S6: Electrospray ionization mass spectrum of **3**; Figure S7: ^1^H NMR spectrum (400 MHz, chloroform-*d*, 298 K) of **4**; Figure S8: ^13^C NMR spectrum (101 MHz, chloroform-*d*, 298 K) of **4**; Figure S9: Electrospray ionization mass spectrum of **4**; Figure S10: ^1^H NMR spectrum (400 MHz, chloroform-*d*, 298 K) of IRCR; Figure S11: Time of flight mass spectrum of IRCR; Figure S12: 2D COSY study of rotaxane IRCR (400 MHz, chloroform-*d*, 298 K); Figure S13: 2D NOESY study of rotaxane IRCR (400 MHz, chloroform-d, 298 K); Figure S14: ^1^H NMR spectrum (400 MHz, DMSO-d6, 298 K) of IR1; Figure S15: Electrospray ionization mass spectrum of IR2; Figure S16: ^1^H NMR spectrum (400 MHz, CD3OD, 298 K) of IR2; Figure S17: ^1^H NMR spectrum (400 MHz, chloroform-*d*, 298 K) of PP5; Figure S18: SEC curve of PP5; Figure S19: Images of the Tyndall effect in water, (a) PP5 (b) PP5-IR1 (c) PP5-IR2 (d) PP5-IR1 after adding GSH; Figure S20: ^1^H NMR spectra (400 MHz, chloroform-*d*, 298 K) (a) IR1 (b) IR1-7th day (c) IR1 + GSH (d) IR1 + GSH-7th day; Figure S21: The ESI-MS of IR1 upon addition of GSH; Figure S22: TEM image (a) and size distribution (b) of PP5-IR1 upon addition of GSH; (c) Particle number distribution for PP5-IR1; (d) Particle number distribution for PP5-IR2; (e) Particle number distribution for PP5-IR1 upon addition of GSH; Figure S23: ^1^H NMR spectra (400 MHz, ((CD_3_)_2_CO/D_2_O = 1:1, 298 K) of IRCR, IRCR+GSH and IRCR+GSH-24 h; Figure S24: 2D NOESY spectrum of IRCR (400 MHz, ((CD_3_)_2_CO/D_2_O = 1:1, 298 K); Figure S25. 2D NOESY spectrum of IRCR upon addition of GSH (400 MHz, ((CD_3_)_2_CO/D_2_O = 1:1, 298 K); Figure S26. (a) Absorption spectra of IR1, IR2, IRCR; (b) fluorescence emission spectra of IR1, IR2, IRCR. (c = 0.40 mg/mL); Figure S27. The emission spectra (λ_ex_ = 808 nm) of IRCR (1.8 mg/mL), PP5-IR1 (0.40 mg/mL), PP5-IR2 (0.40 mg/mL) in water at 7th day; Figure S28. The fluorescence intensity ratio of IRCR (0.36 mg/mL) in CH_3_CN/PBS buffer (2:3, *v*/*v*, 10 mM, pH = 7.4) upon addition of 100 μM various analytes at λ_em_ = 1015 nm:(1) Blank; (2) GSH; (3) Cys; (4) Hcy; (5) Met; (6) Gly; (7) His; (8) Gln; (9) Thr; (10) Asp; (11) Arg; (12) Glu; (13) Phe; (14) Cl^−^; (15) SH^−^; (16) SO_4_^2−^; (17) CO_3_^2−^; (18) pH = 2; Figure S29: The CCK-8 experimental results of A549 incubated with IRCR in EtOH/H_2_O (*v*:*v* = 5:95), PP5-IR1 and PP5-IR2 in H_2_O for 48 h; Scheme S1: Synthesis of PP5. References [39,40,41,42] are cited in the Supplementary Materials.
