# Supplementary material for: Synthesis, Optical Properties and Cellular Toxicity of Water-Soluble near Infrared-II Fluorescent Assemblies Based on Pillar[5]arene
Source: Polymers (Basel). 2023 Sep 21;15(18):3853. doi: 10.3390/polym15183853 (PMC10535555; doi:10.3390/polym15183853)
Supplement: Supplementary file 1 [file polymers-15-03853-s001.zip › polymers-2615618-supplementary.pdf]

Supporting Information

Synthesis, Optical Properties and Cellular Toxicity of Water-soluble NIR-II Fluorescent Assemblies Based on Pillar[5]arene

Qiuxia Wu[1], Xinran Sun[2], Zhenming Yang[1], Pengfei Shi[2], Huacheng Zhang[3,*], and Jie Han[1,*]

[1]State Key Laboratory of Elemento-Organic Chemistry, College of Chemistry, Nankai University, 94 Weijin Road, Tianjin 300071, China. E-mail: hanjie@nankai.edu.cn

[2]Shandong Provincial Key Laboratory of Detection Technology for Tumor Markers, School of Chemistry and Chemical Engineering, Linyi University, Linyi 276000, Shandong, China.

[3]School of Chemical Engineering and Technology, Xi'an Jiaotong University, Xi'an, Shaanxi 710049, China. E-mail: zhanghuacheng@xjtu.edu.cn

1. Synthetic procedures and characterization data……………………..……S2

2. Images of the Tyndall effect of in water…………………………..…….S18

3. Spectra response of IR1 towards GSH……………………………………..S18

4. TEM image and size distribution of **PP5-IR1** upon addition of GSH……S19

5. NMR Spectra response of **IRCR** towards GSH…………………………….S21

6. The optical spectra of **IRCR**, **PP5-IR1**, **PP5-IR2** ……………………..……S23

7. The fluorescence intensity ratio of **IRCR** in CH_3CN/PBS buffer upon addition of various analytes…………………………………………………S24

8. The CCK-8 experimental results of A549 incubated with **IRCR**, **PP5-IR1** and **PP5-IR2** for 48 h…………………………………………………………S24

9. Reference…………………………………………………………………S25

2. Synthetic procedures and characterization data

(1) **Synthesis of 5-(9,9-bis(12-bromododecyl)-9H-fluoren-2-yl)-2,3-dihydrothieno [3,4-b][1,4]dioxine (2)**.[1] **1** (1.84 g, 6.0 mmol) and potassium tert-butanolate (2.02 g, 18.0 mmol) were dissolved in dry THF (40 mL) under nitrogen atmosphere at 0 °C. Then 1,12-dibromododecane (7.88 g, 24.0 mmol) in dry THF (40 mL) was added. After stirring for 6 h at ambient temperature, the reaction was quenched with water. The aqueous phase was extracted three times with dichloromethane, and the combined organic layer was washed with water, brine and dried over $MgSO_4$. Evaporation of the solvent under reduced pressure and further purification was carried out by column chromatography using PE : EA = 40 : 1 as eluent to afford **2** as a light yellow oil (2.04 g, 43%). ^{1}H NMR (400 MHz, Chloroform-*d*) δ 7.73 (d, *J* = 8.0 Hz, 1H), 7.70 – 7.64 (m, 2H), 7.63 (s, 1H), 7.36 – 7.27 (m, 3H), 6.31 (s, 1H), 4.35 – 4.27 (m, 4H), 3.39 (t, *J* = 6.9 Hz, 4H), 2.03 – 1.91 (m, 4H), 1.86 – 1.78 (m, 4H), 1.38 (t, *J* = 7.6 Hz, 4H), 1.27 – 1.01 (m, 28H), 0.70 – 0.52 (m, 4H). ^{13}C NMR (101 MHz, Chloroform-*d*) δ 151.1, 150.9, 142.4, 140.9, 139.7, 138.0, 132.0, 126.9, 126.8, 124.9, 122.9, 120.3, 119.8, 119.6, 118.4, 97.2, 64.8, 64.5, 55.1, 40.4, 34.1, 32.9, 30.1, 29.5, 29.4, 29.3, 28.8, 28.2, 23.8. HRMS (ESI) Calcd. for $C_{43}H_{60}Br_2O_2S$ $[M+H]^+$ 801.2734, found 801.2743.

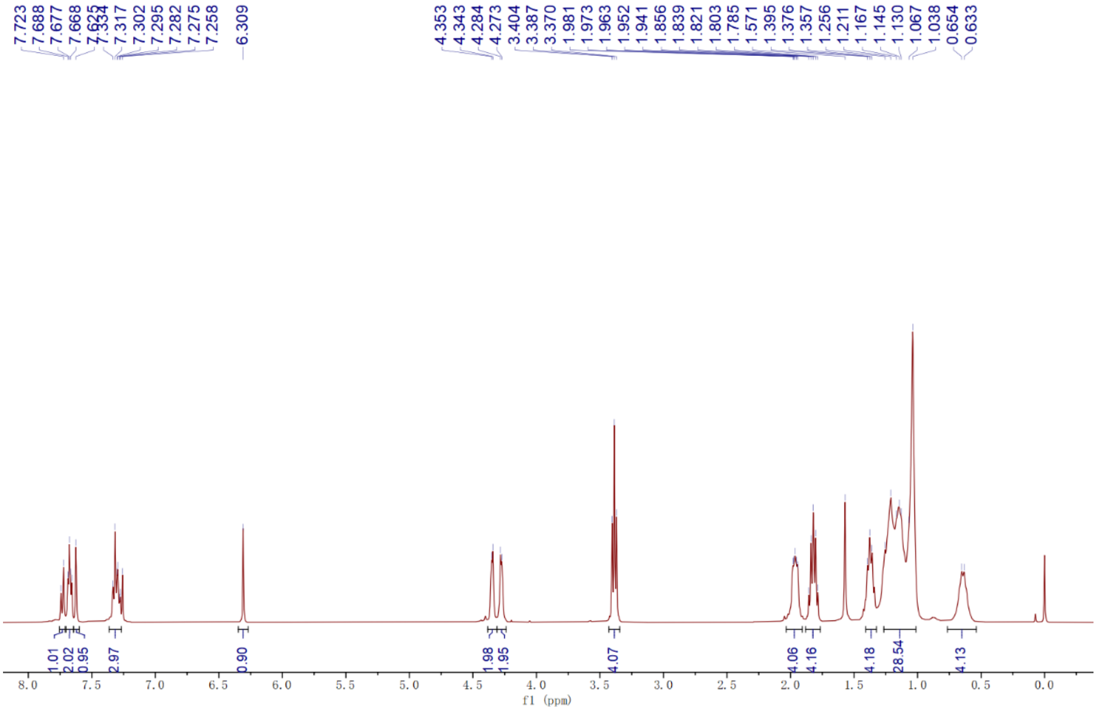

Figure S1. ^{1}H NMR spectrum (400 MHz, chloroform-*d*, 298 K) of **2**

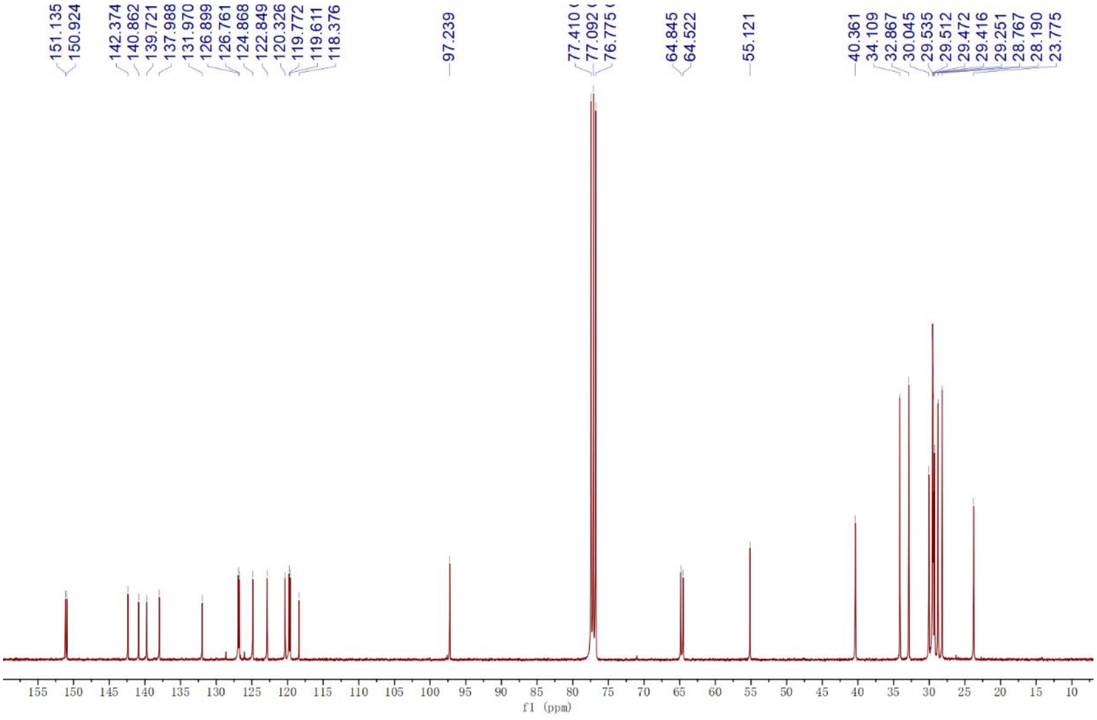

Figure S2. ^{13}C NMR spectrum (101 MHz, chloroform-*d*, 298 K) of **2**

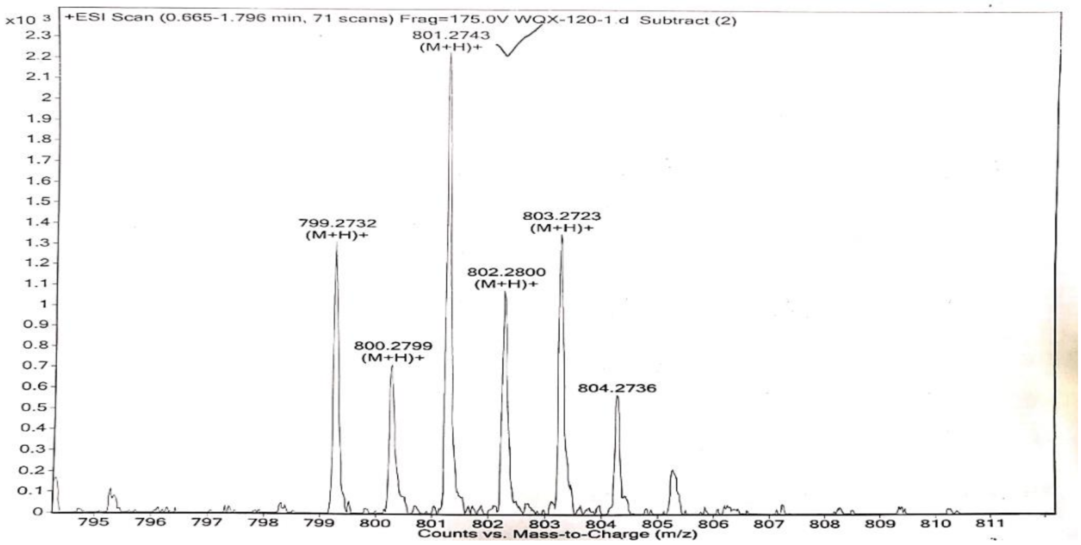

Figure S3. Electrospray ionization mass spectrum of **2**

(2) **Synthesis of (3)**.[1] To a solution of compound **2** (1.06 g, 1.3 mmol) in dry THF (10 mL) at -78 °C under protection gas atmosphere, *n*-BuLi (2.5 M in hexane, 0.6 mL, 1.6 mmol) was added dropwise. After the mixture was stirred at this temperature for another 1.5 h, tributyltinchloride (521 mg, 1.6 mmol) was added to the solution. The reaction mixture was then slowly warmed to room temperature and stirred for 8 h before it was concentrated under reduced pressure. The residue was dissolved in PE, filtered and concentrated to give a yellow oil (9.28 g) without further purification.

To a solution of 4,7-dibroMobenzo[1,2-c:4,5-c']bis([1,2,5]thiadiazole) (1.00 g, 2.8 mmol) and the crude product (9.28 g, 8.5 mmol) in toluene (30 mL) under protection gas atmosphere, $Pd(PPh_3)_2Cl_2$ (598 mg, 0.9 mmol) was added. The mixture was stirred at 120 °C for 12 h. After cooling to room temperature, the mixture was evaporated *in vacuo*. The crude product was subjected to column chromatography using PE : EA = 10 : 1 as eluent to afford **3** as a dark green oil (389 mg, 29%). ^{1}H NMR (400 MHz, Chloroform-*d*) δ 7.92 (d, *J* = 7.9 Hz, 2H), 7.78 (s, 2H), 7.76 – 7.67 (m, 4H), 7.36 – 7.29 (m, 6H), 4.50 (s, 4H), 4.37 (s, 4H), 3.36 (t, *J* = 6.9 Hz, 8H), 2.08 – 1.94 (m, 8H), 1.83 – 1.75 (m, 8H), 1.37– 1.32 (m, 8H), 1.31 – 0.94 (m, 64H), 0.71 – 0.63 (m, 8H). ^{13}C NMR (101 MHz, Chloroform-

d) δ 152.6, 151.1, 142.0, 140.8, 140.4, 138.4, 131.7, 128.0, 127.7, 126.4, 126.1, 124.7, 123.7, 122.9, 122.1, 121.6, 120.7, 120.5, 120.0, 119.1, 113.2, 108.8, 66.2, 64.8, 64.6, 63.3, 55.2, 41.7, 40.5, 39.2, 35.6, 34.1, 32.9, 31.6, 31.4, 30.7, 30.1, 29.5, 28.8, 28.2, 25.1, 23.9, 22.6. HRMS (ESI) Calcd. for $C_{92}H_{118}Br_4N_4O_4S_4$ $[M+H]^+$ 1791.4802, found 1791.4835.

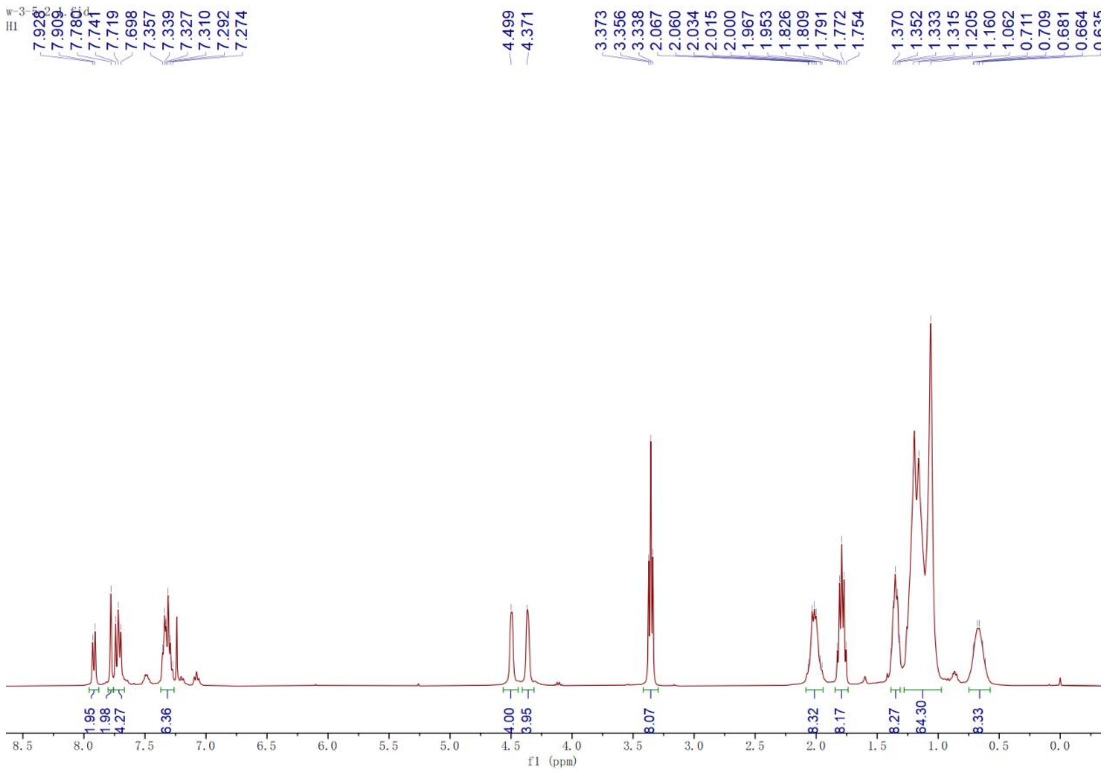

Figure S4. ^{1}H NMR spectrum (400 MHz, chloroform-*d*, 298 K) of **3**

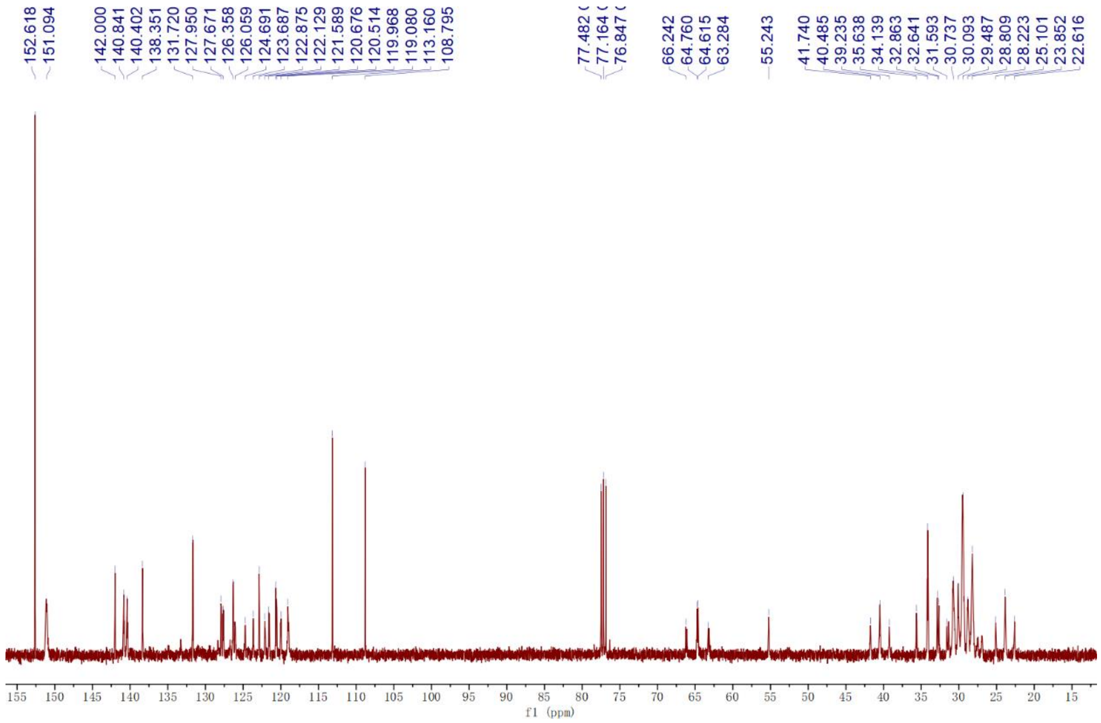

Figure S5. ^{13}C NMR spectrum (101 MHz, chloroform-*d*, 298 K) of **3**

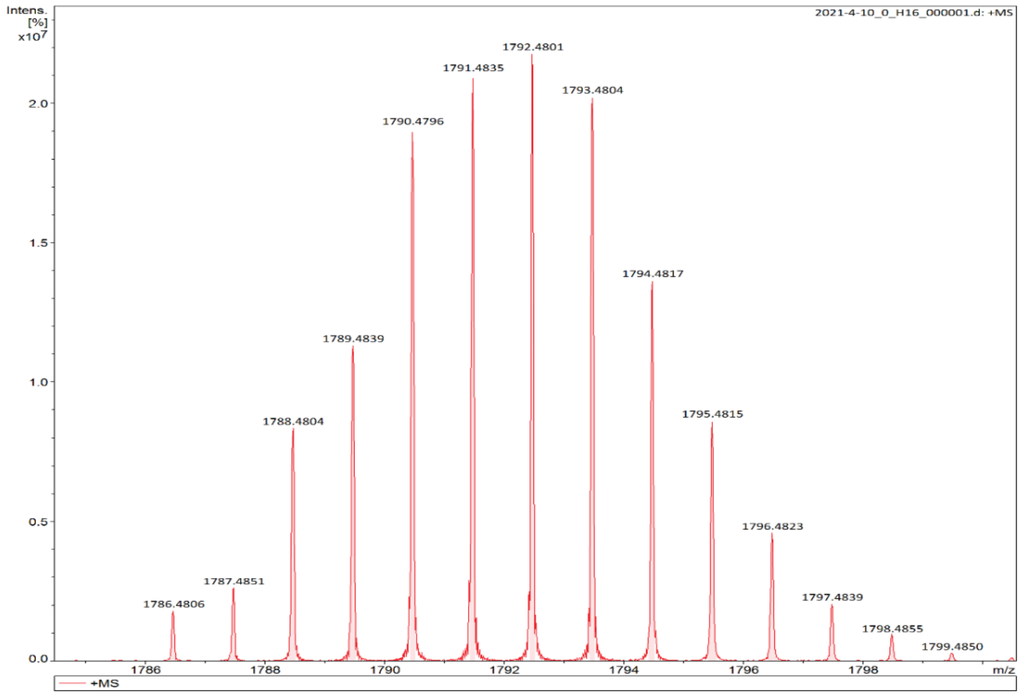

Figure S6. Electrospray ionization mass spectrum of **3**

(3) **Synthesis of (4).**[2] To a solution of 4-picoline (996 mg, 10.7 mmol) in dry THF (10 mL) at -78 °C under protection gas atmosphere, lithium diisopropyl amide (2.0 M, 5.4 mL, 10.7 mmol) was added dropwise. After the mixture was

stirred at this temperature for another 1.5 h, compound **3** (1.92 g, 1.1 mmol) in dry THF (20 mL) was added to the solution. The reaction mixture was then slowly warmed to room temperature and stirred for 24 h before it was concentrated under reduced pressure. The crude product was subjected to column chromatography using DCM : MeOH = 40 : 1 as eluent to afford **4** as a dark green oil (1.42 g, 72%). ^{1}H NMR (400 MHz, Chloroform-*d*) δ 8.47 (d, *J* = 5.2 Hz, 8H), 7.93 (d, *J* = 7.9 Hz, 2H), 7.79 – 7.72 (m, 6H), 7.37 – 7.28 (m, 6H), 7.09 (d, *J* = 5.3 Hz, 8H), 4.52 (s, 4H), 4.39 (s, 4H), 2.57 (t, *J* = 7.6 Hz, 8H), 2.08 – 1.95 (m, 8H), 1.63 – 1.57 (m, 8H), 1.32– 0.98 (m, 64H), 0.71 – 0.63 (m, 8H). 0.70 – 0.60 (m, 8H). ^{13}C NMR (101 MHz, Chloroform-*d*) δ 152.6, 151.8, 151.2, 151.0, 149.9, 149.6, 149.4, 142.0, 140.8, 140.4, 138.3, 131.6, 127.1, 126.8, 125.5, 123.9, 122.9, 121.1, 120.8, 119.8, 119.7, 113.1, 108.8, 64.7, 64.6, 55.2, 40.5, 35.2, 30.3, 30.1, 29.6, 29.5, 29.4, 29.3, 29.2, 23.8. HRMS (ESI) Calcd. for $C_{116}H_{142}N_8O_4S_4$ $[M+H]^+$ 1841.0144, found 1841.0167.

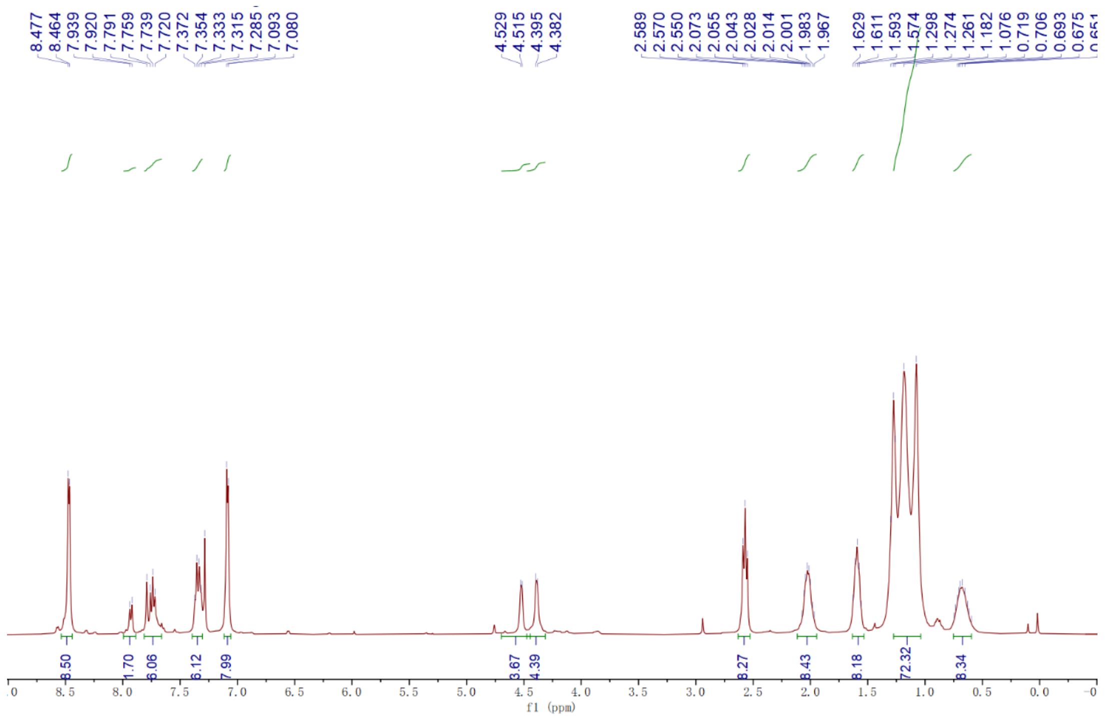

Figure S7. ^{1}H NMR spectrum (400 MHz, chloroform-*d*, 298 K) of **4**

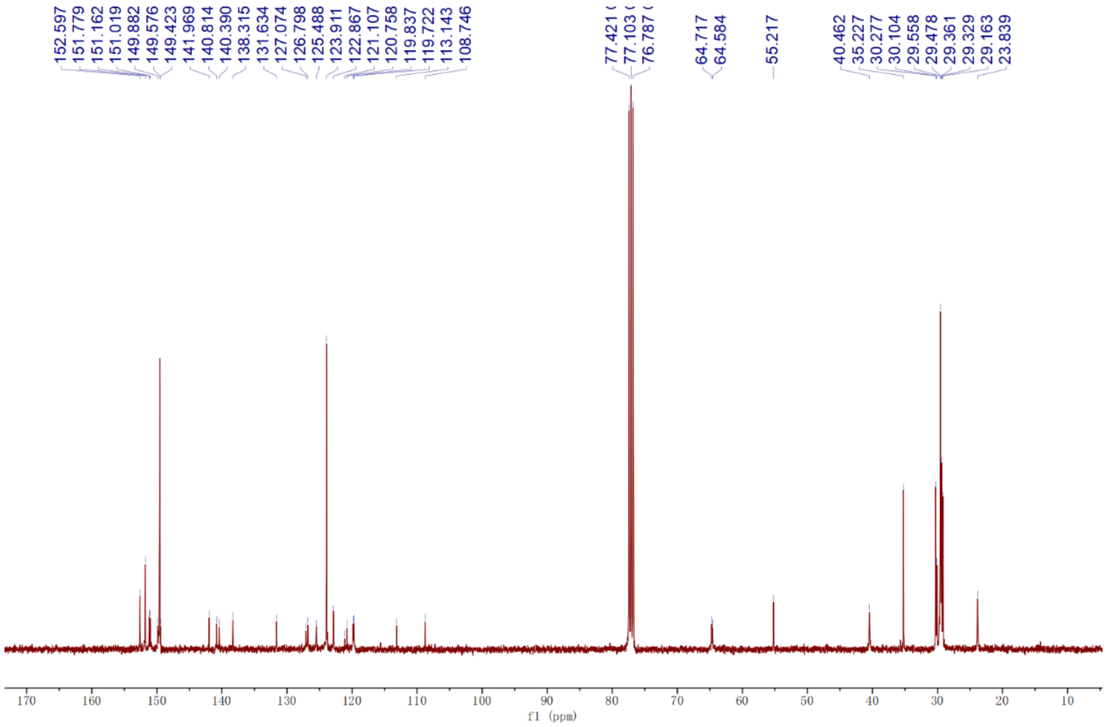

Figure S8. ^{13}C NMR spectrum (101 MHz, chloroform-*d*, 298 K) of **4**

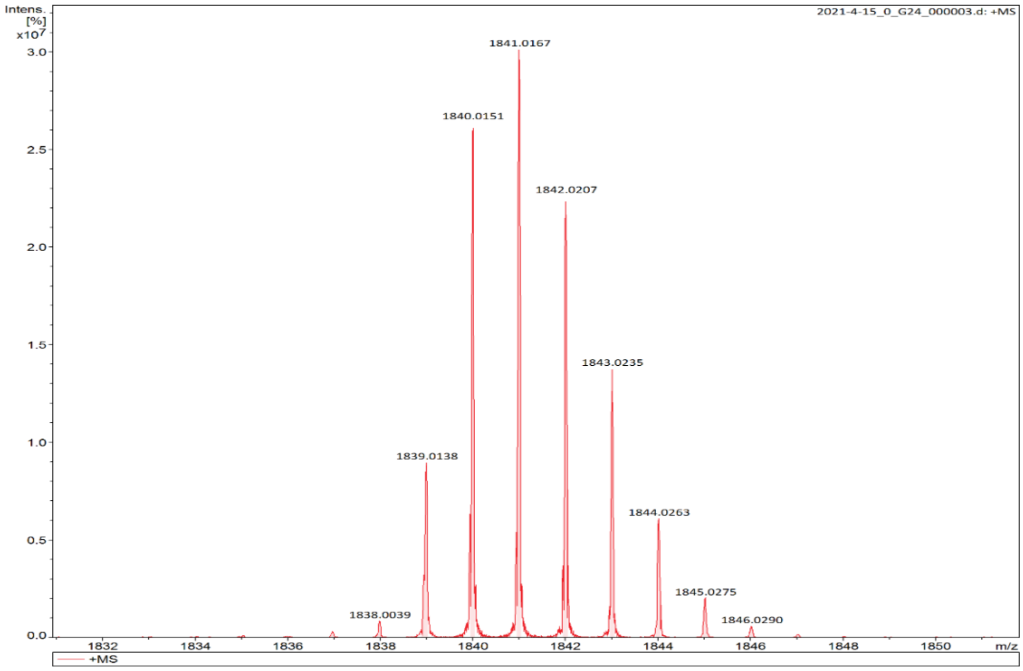

Figure S9. Electrospray ionization mass spectrum of **4**

(4) **Synthesis of IRCR.**[3] Compound **4** (447 mg, 0.24 mmol), 1-(4-(bromomethyl) phenoxy)-2,4-dinitrobenzene[4] (685 mg, 1.94 mmol) were dissolved in Cyclic Host Liquids[5] (**CHL**, 12.43 g, 6.00 mmol), and the mixture was stirred for 24 h at 70 °C. The crude product was directly subjected to

column chromatography using DCM : MeOH = 25:1 as eluent to afford **IRCR** as a dark green oil (2.18 g, 79%).

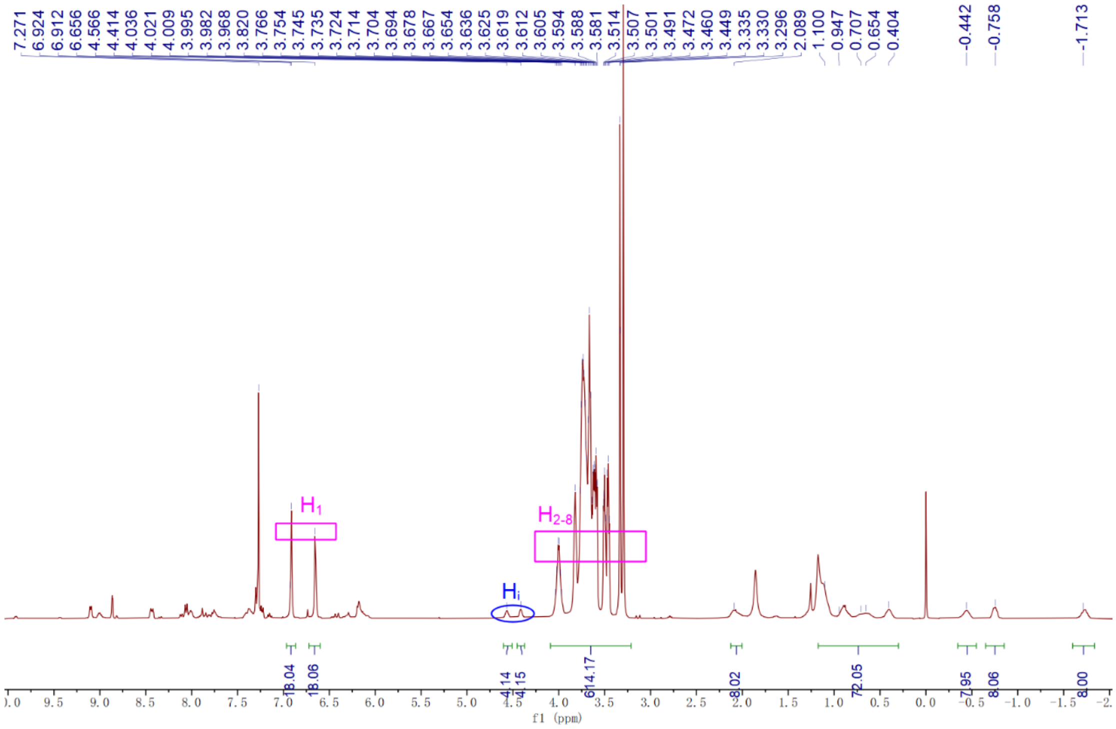

Figure S10. ^{1}H NMR spectrum (400 MHz, chloroform-*d*, 298 K) of **IRCR**

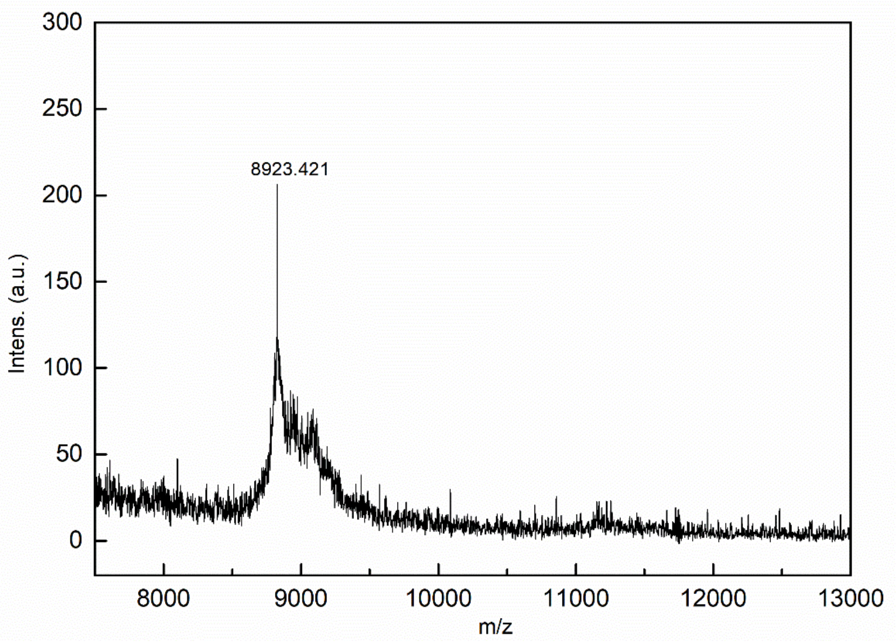

Figure S11. Time of flight mass spectrum of **IRCR**

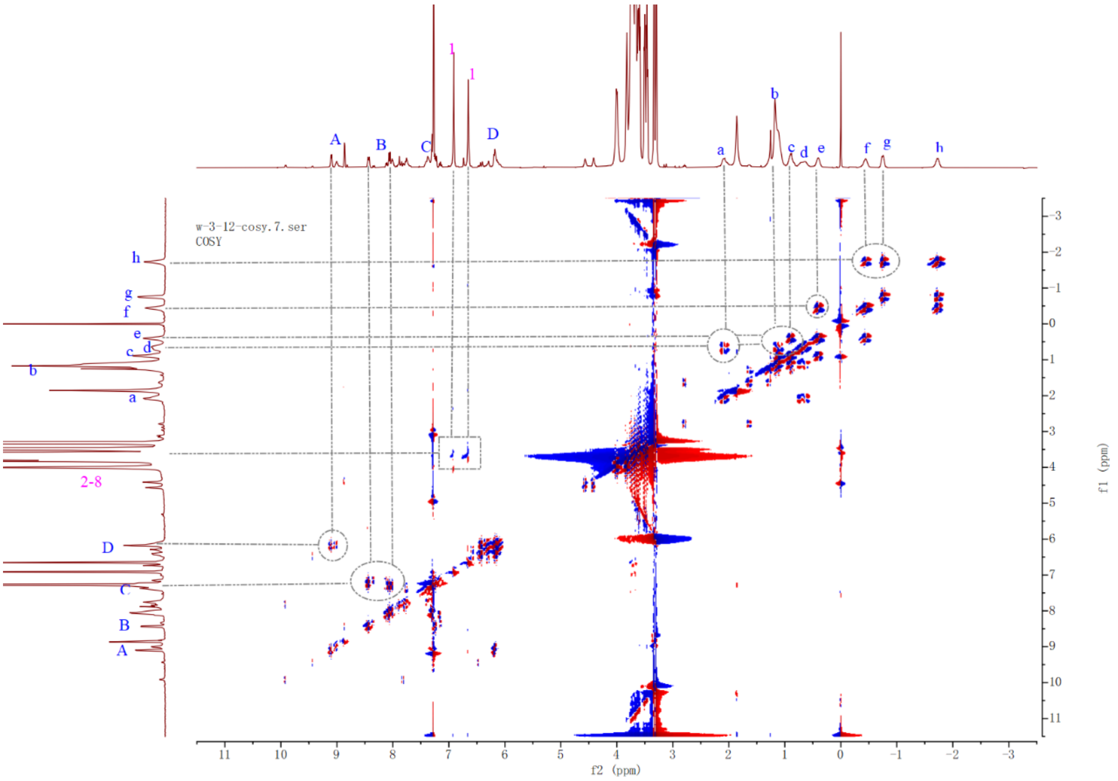

Figure S12. 2D COSY study of rotaxane **IRCR** (400 MHz, chloroform-*d*, 298 K)

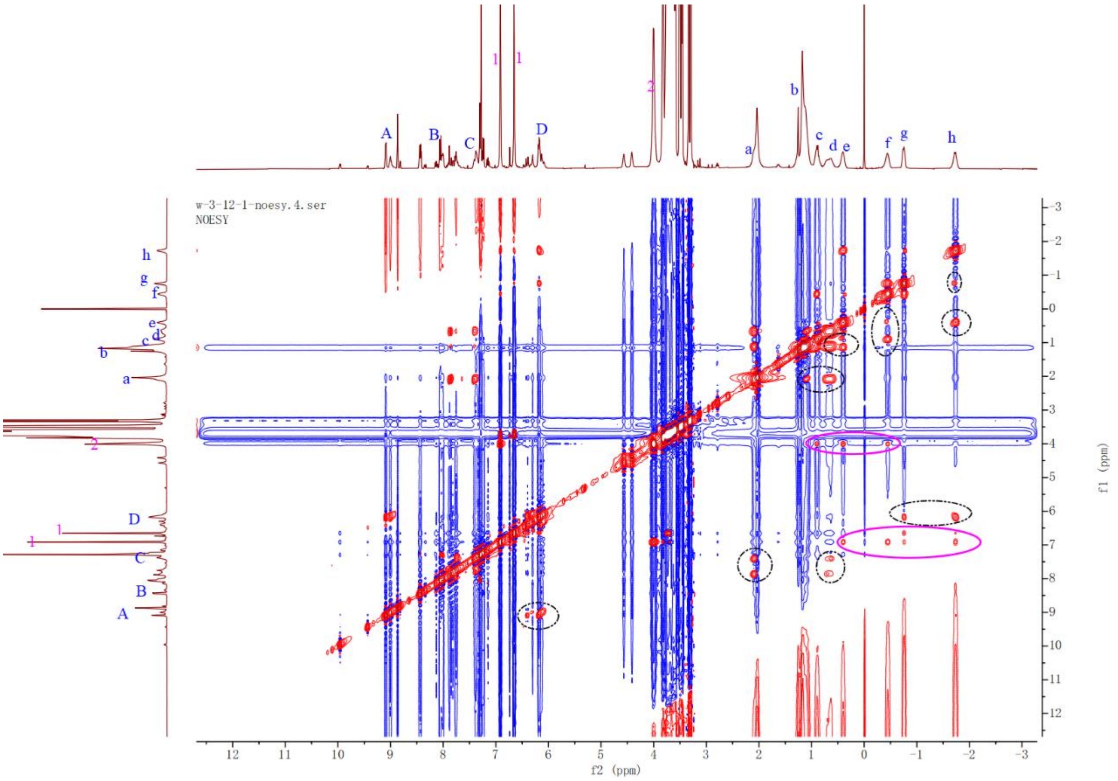

Figure S13. 2D NOESY study of rotaxane **IRCR** (400 MHz, chloroform-*d*, 298 K)

(5) **Synthesis of IR1**. Compound **4** (117 mg, 0.06 mmol), 1-(4-(bromomethyl) phenoxy)-2,4-dinitrobenzene (177 mg, 0.50 mmol) were dissolved in toluene (5 mL), and the reaction mixture was stirred under reflux for 12 h. After decanting the solvent, solid compound sticking to the wall of the glass bottle was dissolved in CH_3OH, and dried firstly in the rotator and then at high vacuum. ^1H NMR (400 MHz, DMSO-d_6) δ 9.12 – 9.08 (m, 6H), 8.94 – 8.90 (m, 4H), 8.46 – 8.43 (m, 6H), 8.05 – 8.02 (m, 8H), 7.94 – 7.74 (m, 8H), 7.70 – 7.69 (m, 8H), 7.38 – 7.32 (m, 12H), 7.23 – 7.19 (m, 6H), 5.82 (s, 8H), 4.50 (s, 4H), 4.37 (s, 4H), 2.84 – 2.80 (m, 8H), 2.11 – 1.88 (m, 8H), 1.60 – 1.54 (m, 8H), 1.36 – 0.83 (m, 72H), 0.64 – 0.50 (m, 8H).

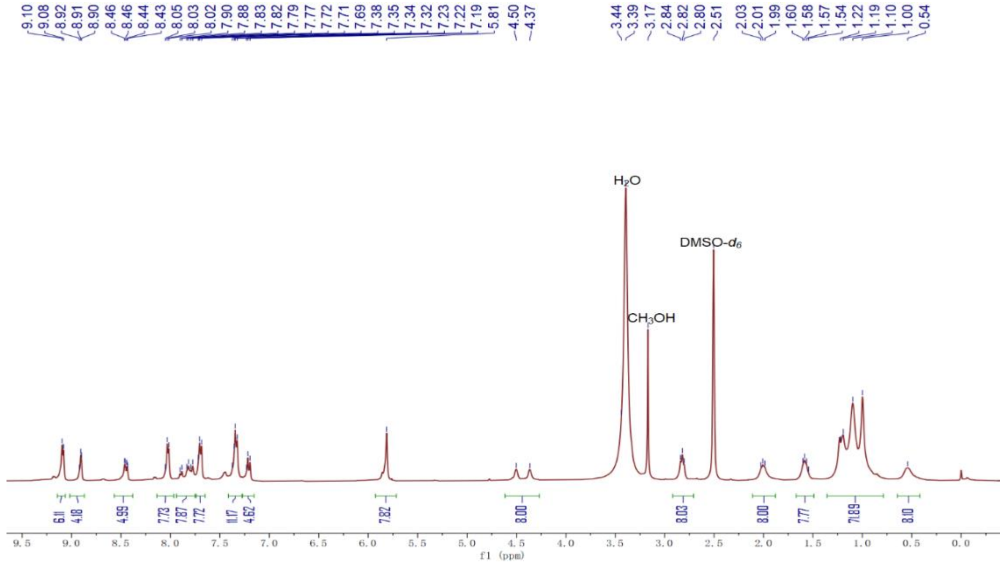

Figure S14. ^1H NMR spectrum (400 MHz, DMSO-d_6, 298 K) of **IR1**

(6) **Synthesis of IR2.** Compound **4** (117 mg, 0.06 mmol), bromoethane (55 mg, 0.50 mmol) were dissolved in toluene (5 mL), and the reaction mixture was stirred under nitrogen atmosphere at 70 °C for 48 h. After decanting the solvent, solid compound sticking to the wall of the glass bottle was dissolved in CH_3OH, and dried firstly in the rotator and then at high vacuum. ^1H NMR (400 MHz, Methanol-d_4) δ 8.81 (m, 8H), 8.06 – 7.78 (m, 16H), 7.35 (m, 6H), 4.65 – 4.54 (m, 12H), 4.44 (m, 4H), 2.85 (m, 8H), 2.08 (m, 8H), 1.62 (m, 20H), 1.12 (m,

72H), 0.63 (m, 8H). HRMS (ESI) Calcd. for $C_{124}H_{162}N_8O_4S_4$ [M+1] 1955.1580, found 1954.1502.

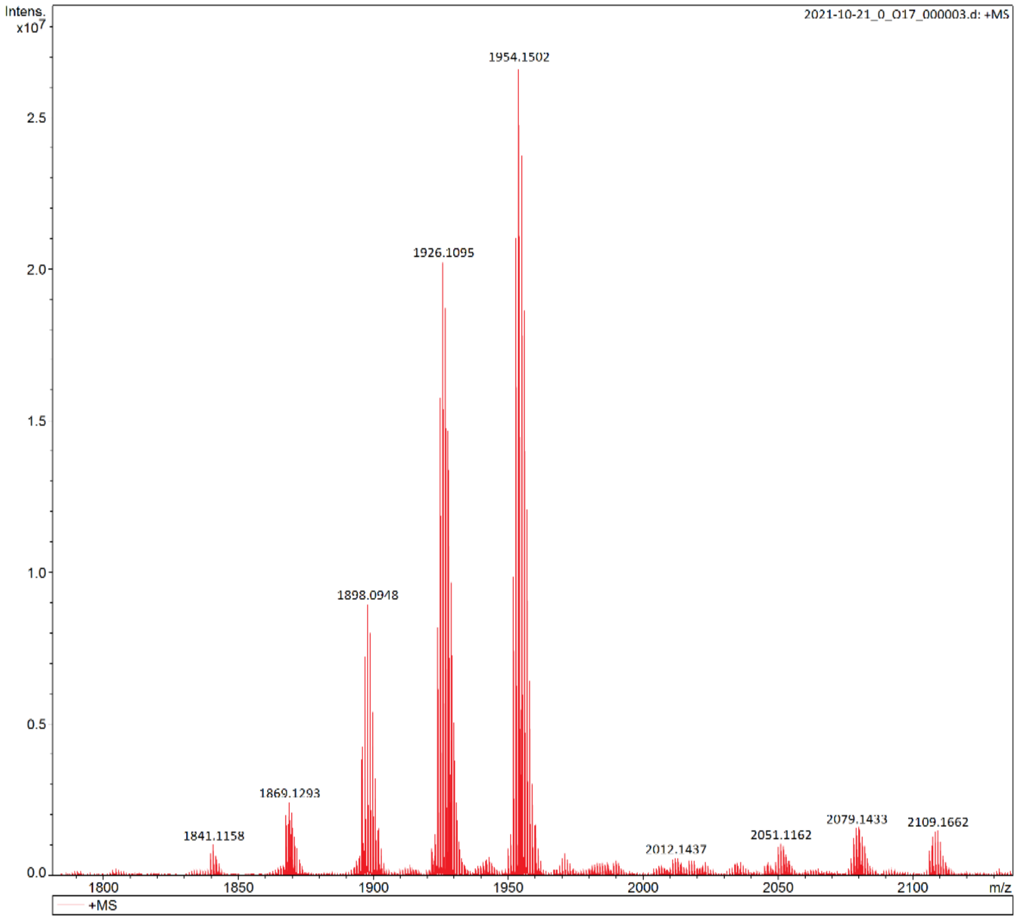

Figure S15. Electrospray ionization mass spectrum of **IR2**

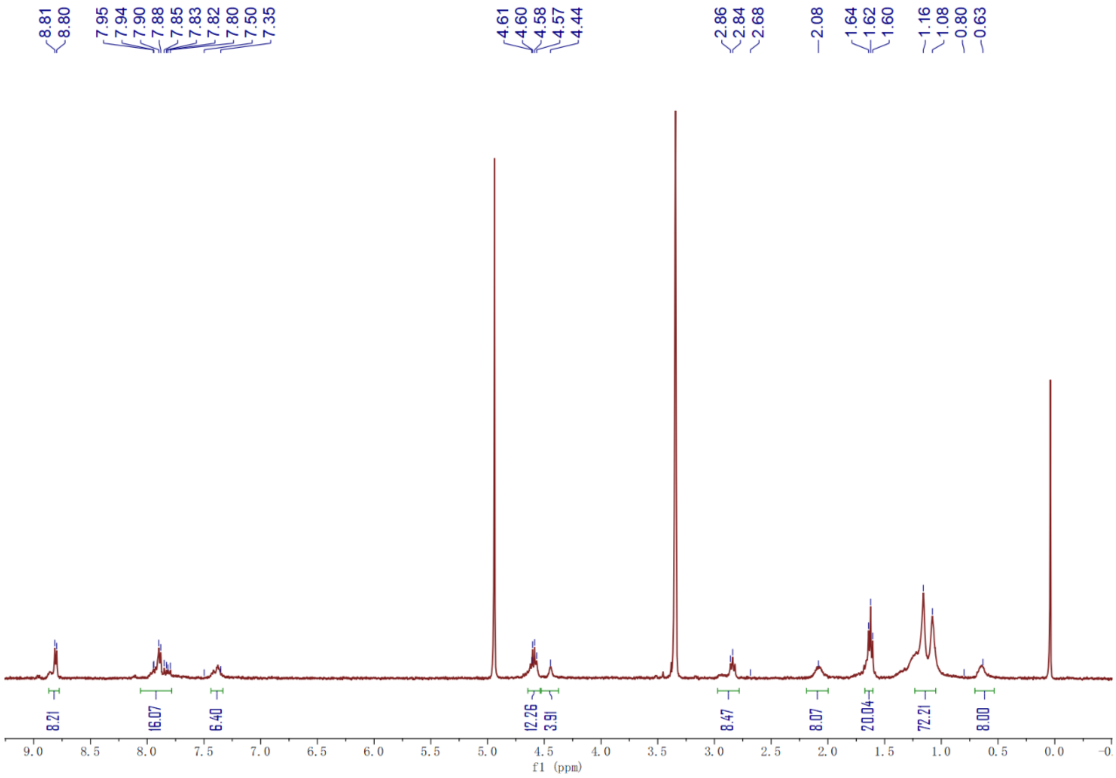

Figure S16. ^{1}H NMR spectrum (400 MHz, CD_3OD, 298 K) of **IR2**

(7) **Synthesis of PP5**[6]

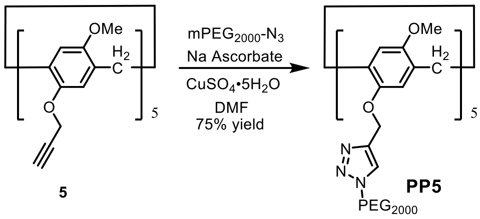

Scheme S1. Synthesis of **PP5**

$mPEG_{2000}$-N_3 (1.00 g, 0.5 mmol), copper sulfate pentahydrate (3 mg, 0.013 mmol) and sodium ascorbate (5 mg, 0.025 mmol) were added to a solution of compound **5** (74 mg, 0.085 mmol) in *N N′*-dimethylformamide (10 mL). The mixture was heated in a three-necked flask under nitrogen atmosphere at 90 °C for 1 d. The reaction mixture was diluted with dichloromethane and washed with water. The organic phase was dried over magnesium sulfate and filtered. The solvent was removed under reduced pressure. The residue was dissolved in THF and precipitated into an excess of cold diethyl ether. The

above dissolution-precipitation cycle was repeated three times. The final product was dried in a vacuum oven overnight at room temperature, yielding a white solid (667 mg, 75%). The molecular composition and weight of **PP5** were determined by ^{1}H NMR spectrum (**Figure S18**) and SEC curve (**Figure S19**).

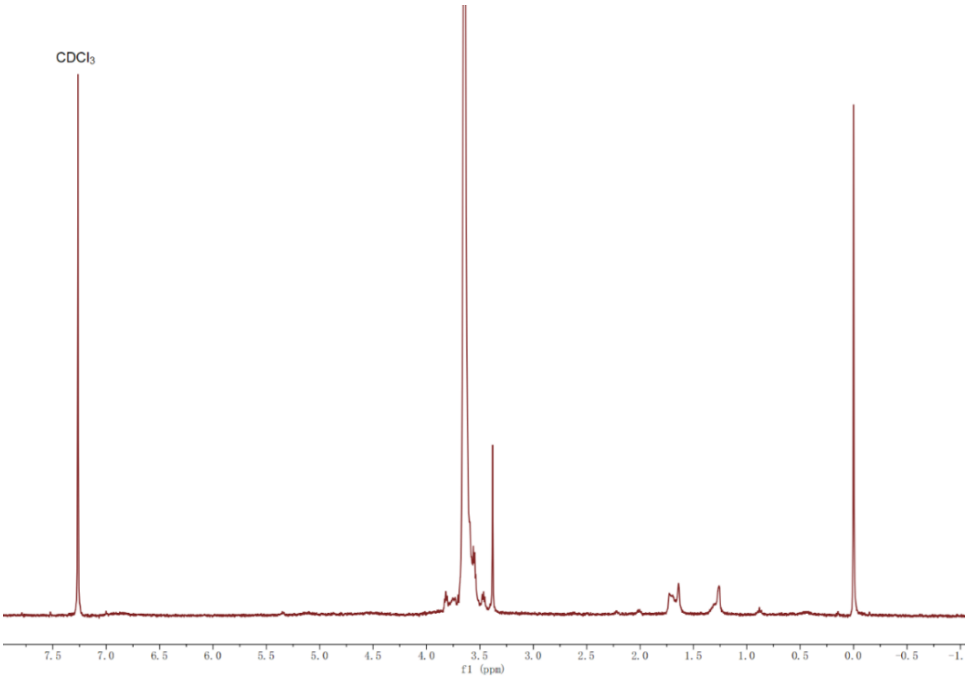

Figure S17. ^{1}H NMR spectrum (400 MHz, chloroform-*d*, 298 K) of **PP5**

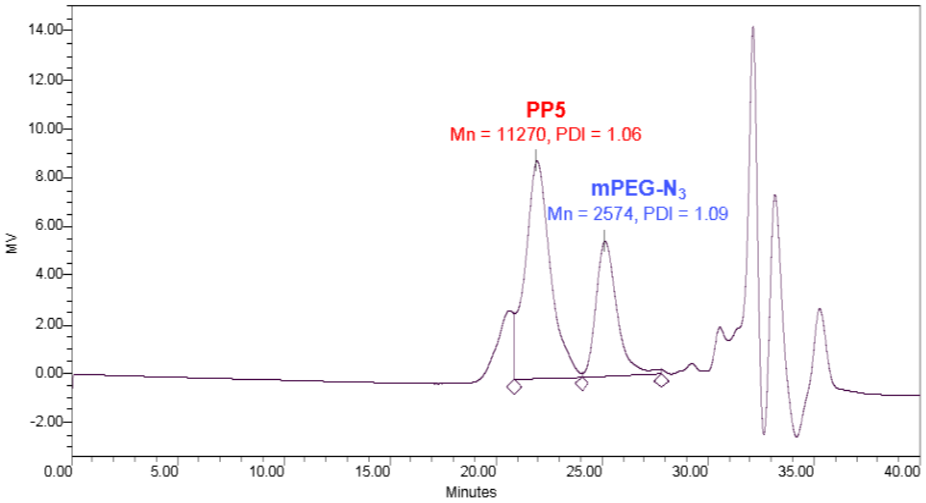

Figure S18. SEC curve of **PP5**

2. Images of the Tyndall effect in water

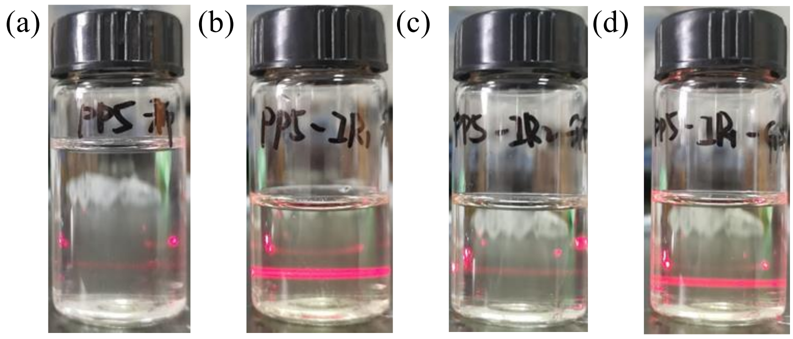

Figure S19. Images of the Tyndall effect in water, (a) **PP5** (b) **PP5-IR1** (c) **PP5-IR2** (d) **PP5-IR1** after adding GSH

3. Spectra response of IR1 towards GSH

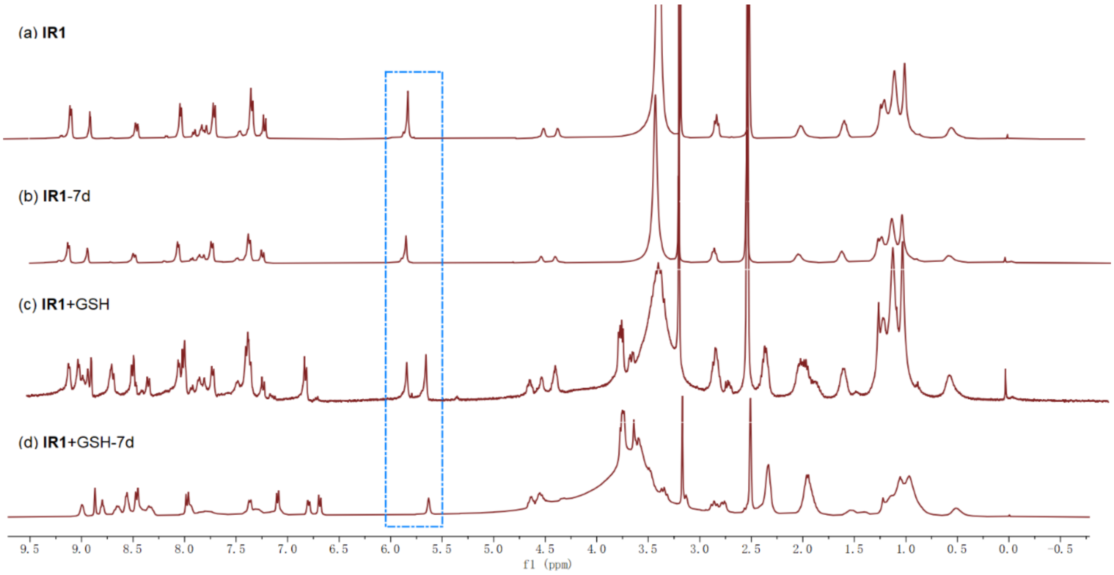

Figure S20. ^{1}H NMR spectra (400 MHz, chloroform-*d*, 298 K) (a) **IR1** (b) **IR1**-7th day (c) **IR1**+GSH (d) **IR1**+GSH-7th day

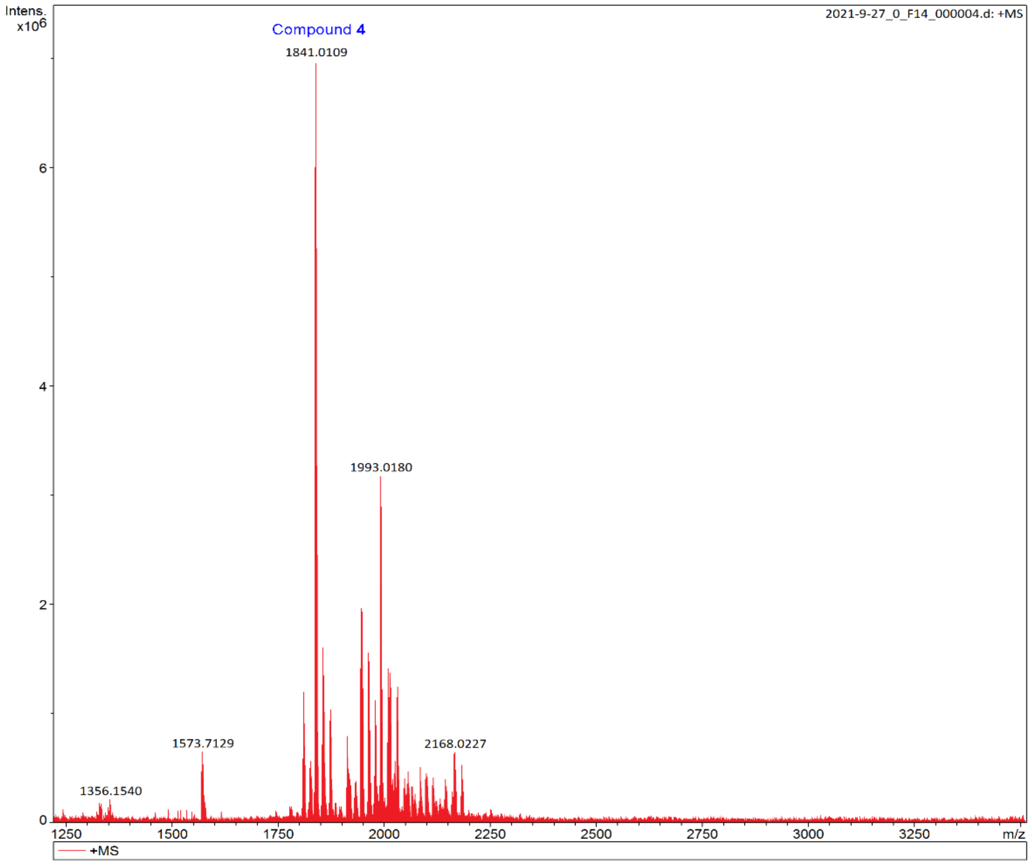

Figure S21. The ESI-MS of **IR1** upon addition of GSH

4. TEM image and size distribution of PP5-IR1 upon addition of GSH.

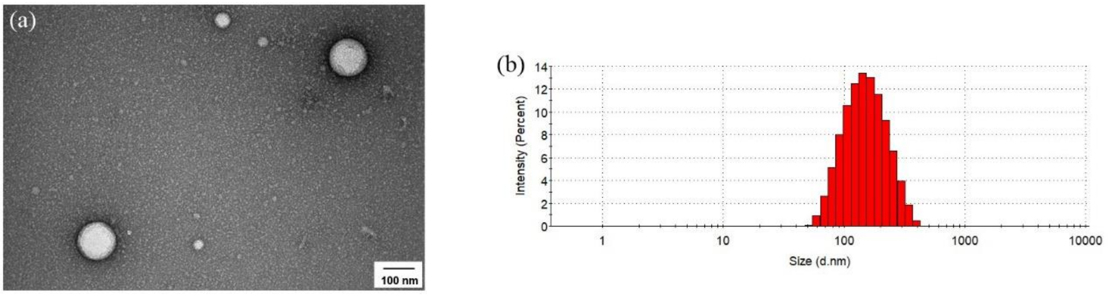

Figure S22. TEM image (a) and size distribution (b) of PP5-IR1 upon addition of GSH.

			Size (d.nm):	% Number:	St Dev (d.nm):
Z-Average (d.nm):	153.9	Peak 1:	99.40	100.0	32.24
PdI:	0.119	Peak 2:	0.000	0.0	0.000
Intercept:	0.913	Peak 3:	0.000	0.0	0.000

Result quality : Good

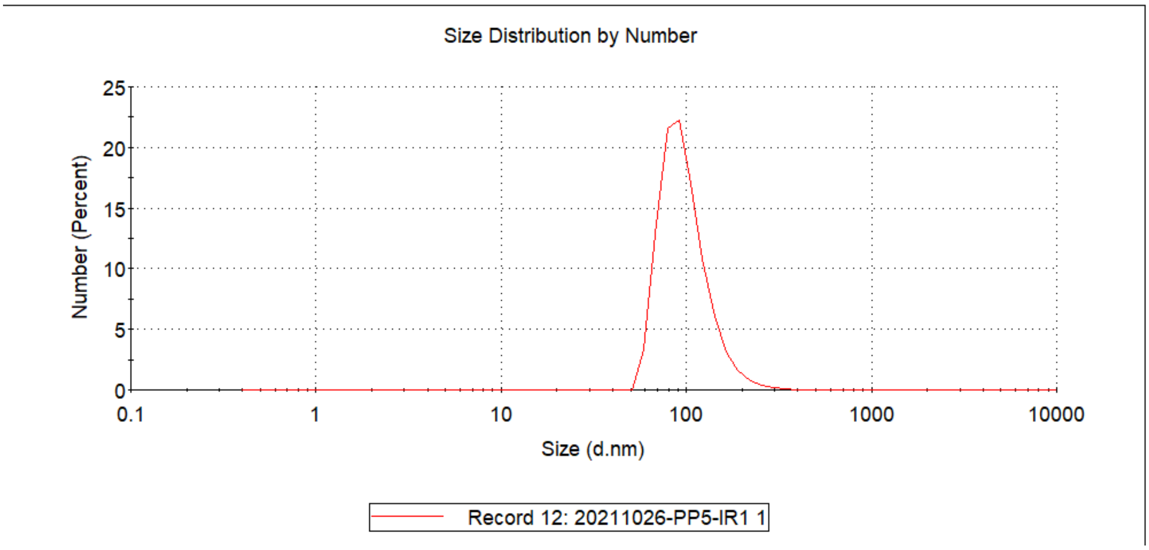

Figure S22c. Particle number distribution for PP5-IR1

			Size (d.nm):	% Number:	St Dev (d.nm):
Z-Average (d.nm):	82.00	Peak 1:	48.93	100.0	14.75
PdI:	0.183	Peak 2:	0.000	0.0	0.000
Intercept:	0.749	Peak 3:	0.000	0.0	0.000

Result quality : Good

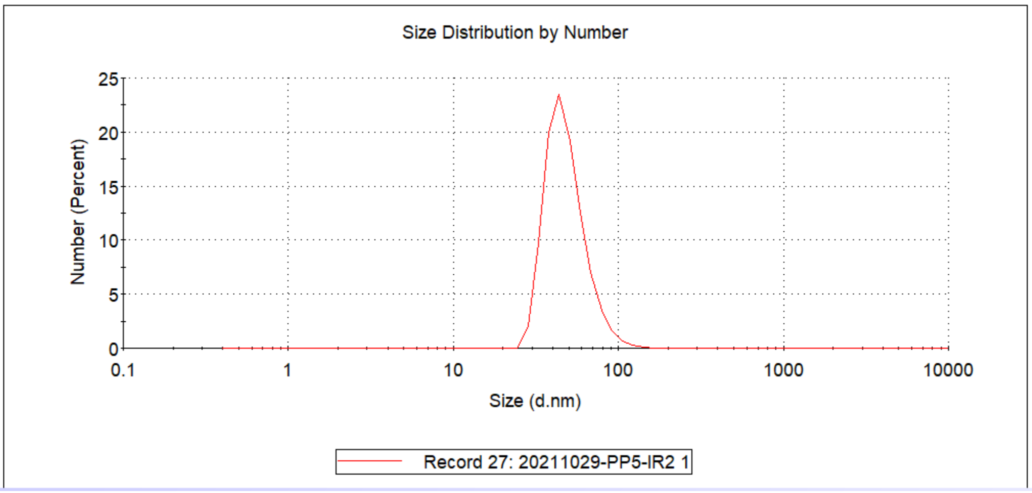

Figure S22d. Particle number distribution for PP5-IR2

			Size (d.nm):	% Number:	St Dev (d.nm):
Z-Average (d.nm):	132.7	Peak 1:	80.37	100.0	26.13
PdI:	0.163	Peak 2:	0.000	0.0	0.000
Intercept:	0.921	Peak 3:	0.000	0.0	0.000
Result quality :	Good				

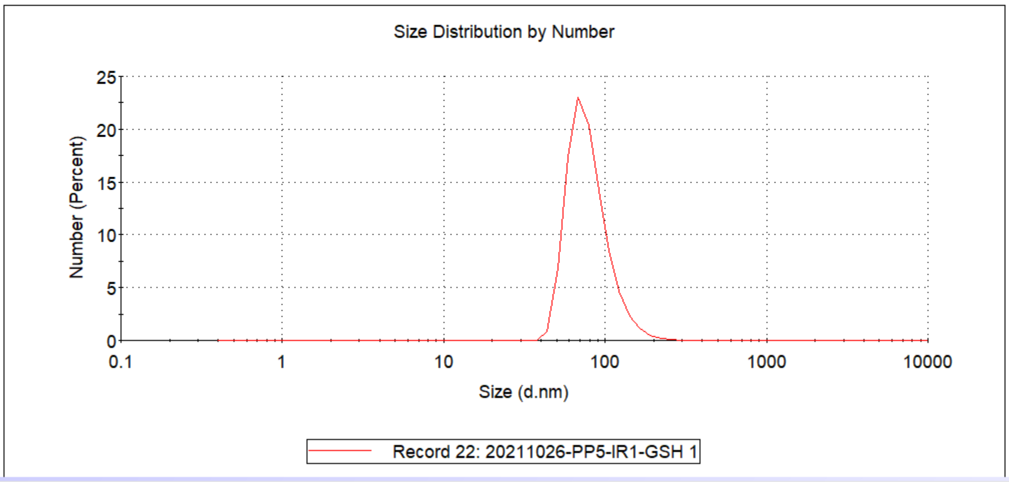

Figure S22e. Particle number distribution for PP5-IR1 upon addition of GSH.

5. NMR spectra response of IRCR towards GSH

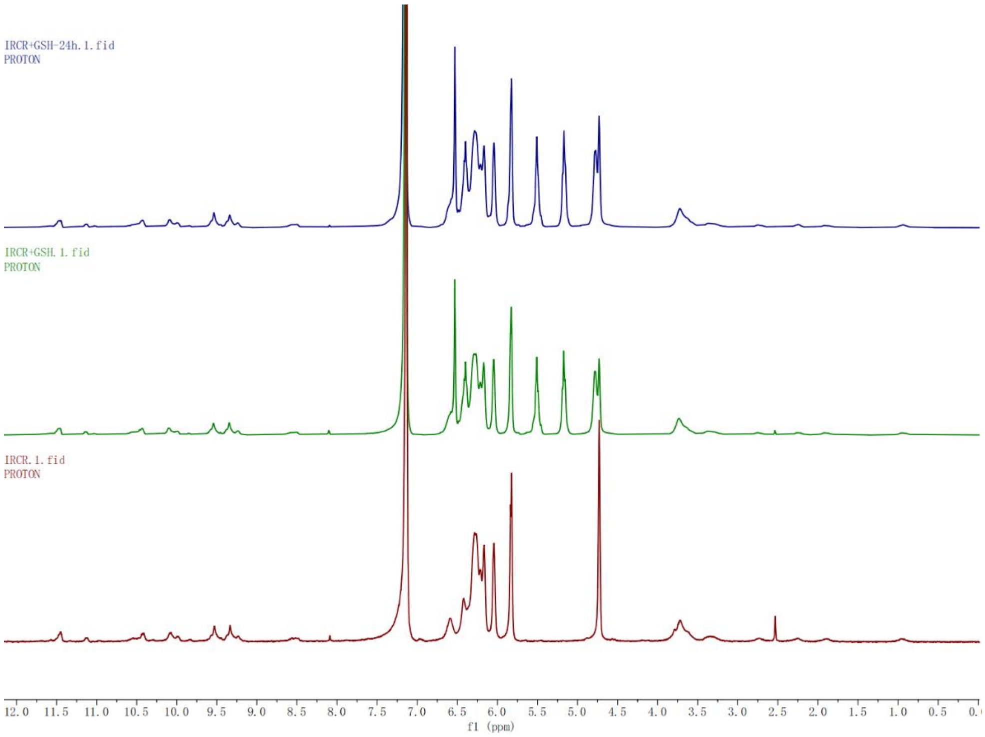

Figure S23. ^{1}H NMR spectra (400 MHz, $((CD_3)_2CO/D_2O$ = 1:1, 298 K) of **IRCR**, **IRCR**+GSH and **IRCR**+GSH-24 h

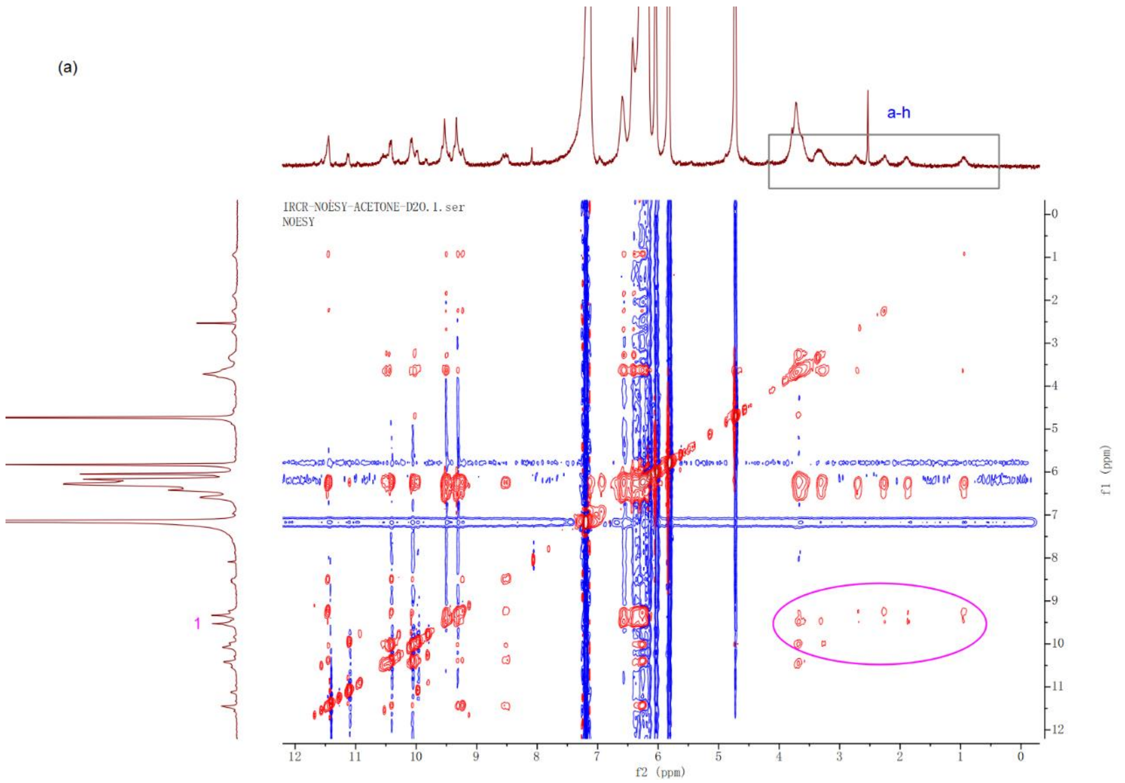

Figure S24. 2D NOESY spectrum of **IRCR** (400 MHz, $((CD_3)_2CO/D_2O = 1:1$, 298 K)

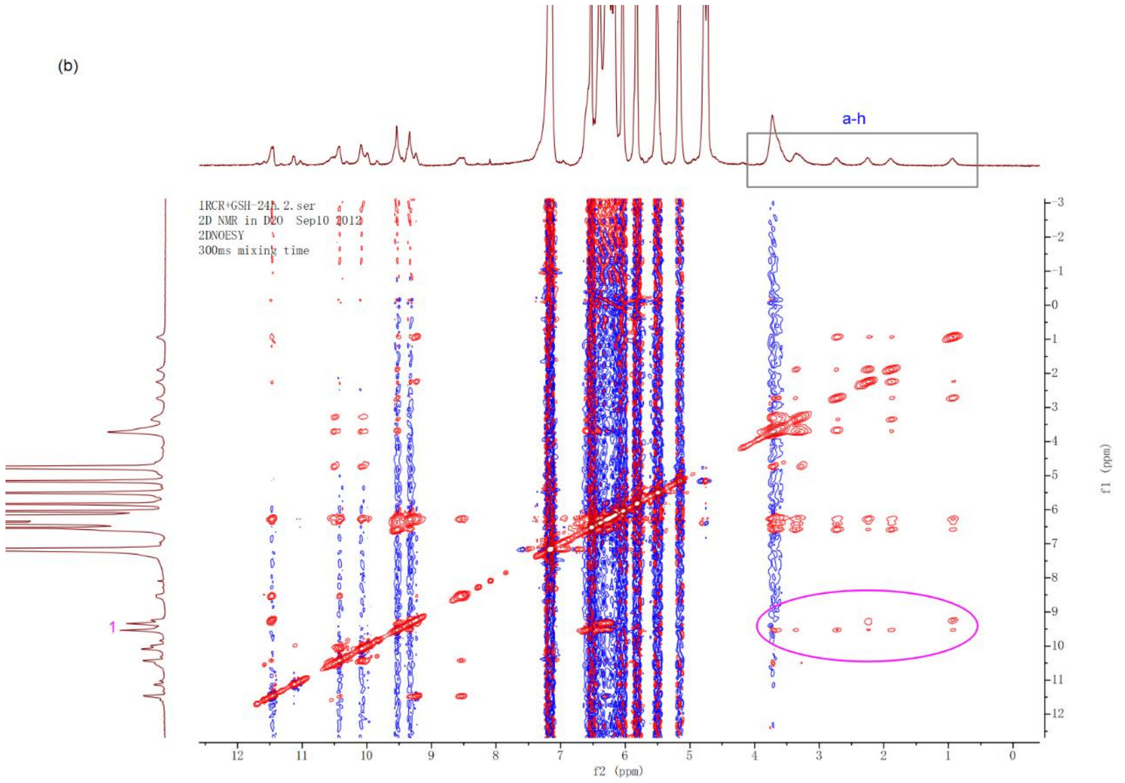

Figure S25. 2D NOESY spectrum of **IRCR** upon addition of GSH (400 MHz, $((CD_3)_2CO/D_2O = 1:1$, 298 K)

6. The optical spectra of IRCR, PP5-IR1, PP5-IR2

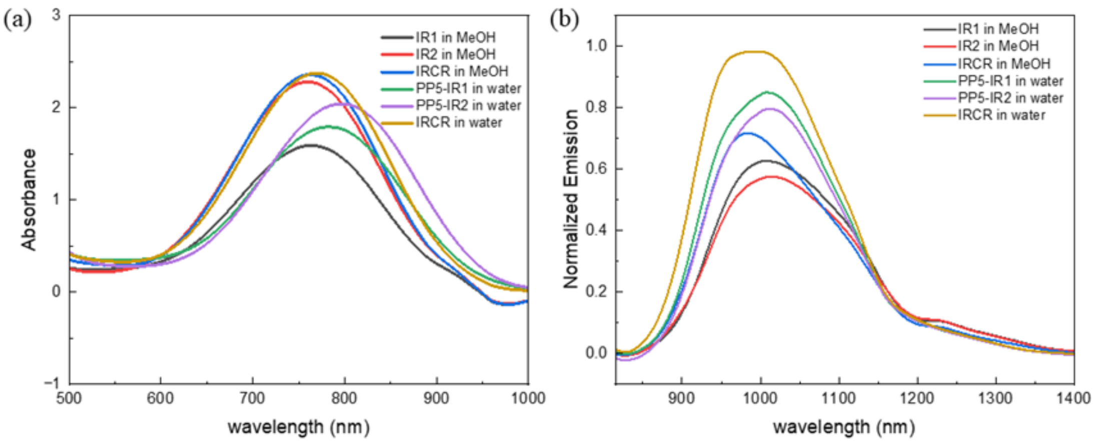

Figure S26. (a) Absorption spectra of **IR1**, **IR2**, **IRCR**; (b) fluorescence emission spectra of **IR1**, **IR2**, **IRCR**. (c = 0.40 mg/mL).

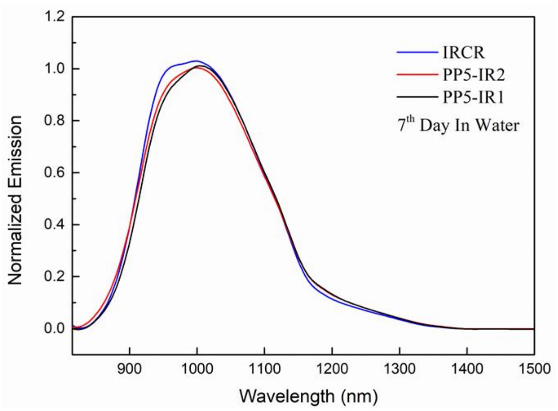

Figure S27. The emission spectra (λ_{ex} = 808 nm) of **IRCR** (1.8 mg/mL), **PP5-IR1** (0.40 mg/mL), **PP5-IR2** (0.40 mg/mL) in water at 7th day

7. The fluorescence intensity ratio of IRCR in CH_3CN/PBS buffer upon addition of various analytes

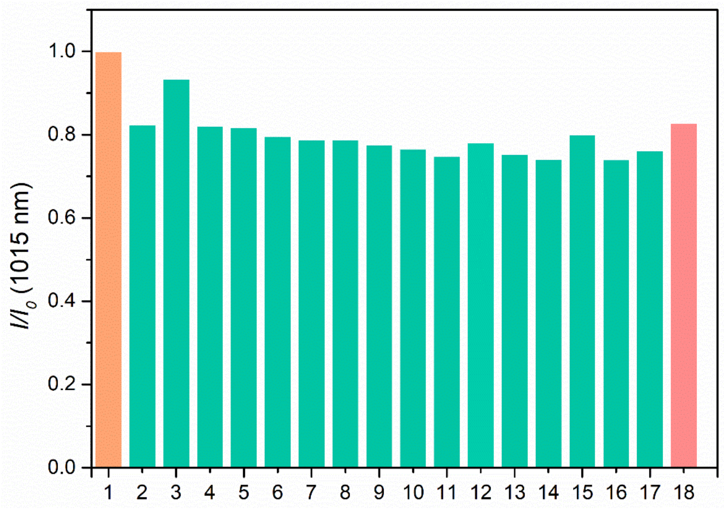

Figure S28. The fluorescence intensity ratio of **IRCR** (0.36 mg/mL) in CH_3CN/PBS buffer (2:3, v/v, 10 mM, pH = 7.4) upon addition of 100 μM various analytes at λ_{em} = 1015 nm:(1) Blank; (2) GSH; (3) Cys; (4) Hcy; (5) Met; (6) Gly; (7) His; (8) Gln; (9) Thr; (10) Asp; (11) Arg; (12) Glu; (13) Phe; (14) Cl^-; (15) SH^-; (16) SO_4^{2-}; (17) CO_3^{2-}; (18) pH = 2.

8. The CCK-8 experimental results of A549 incubated with IRCR, PP5-IR1 and PP5-IR2 for 48 h

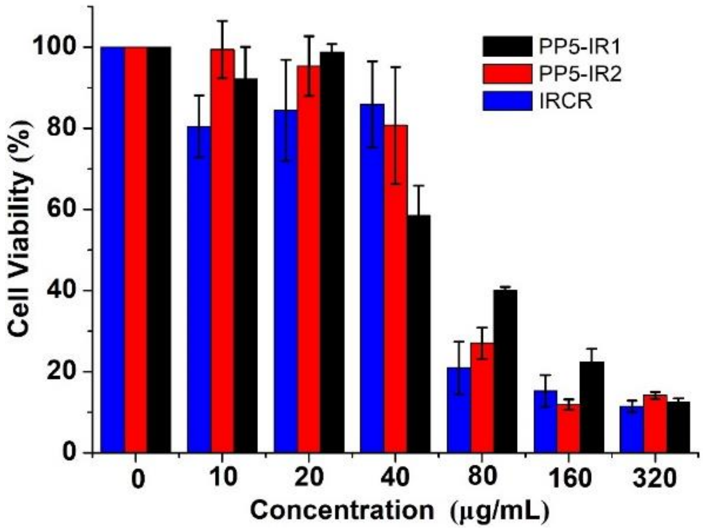

Figure S29. The CCK-8 experimental results of A549 incubated with **IRCR** in EtOH/H_2O (v:v = 5:95), **PP5-IR1** and **PP5-IR2** in H_2O for 48 h.

9. References

[1] Q. Yang, Z. Ma, H. Wang, B. Zhou, S. Zhu, Y. Zhong, J. Wang, H. Wan, A. Antaris, R. Ma, X. Zhang, J. Yang, X. Zhang, H. Sun, W. Liu, Y. Liang, H. Dai, *Adv. Mater.* **2017**, *29*, 1605497.

[2] A. I. M. C. Lobo Ferreira, A. S. M. C. Rodrigues, M. Villas, E. Tojo, L. P. N. Rebelo, L. M. N. B. F. Santos, *ACS Sustainable Chem. Eng.* **2019**, *7*, 2989-2997.

[3] T. Ogoshi, T. Aoki, R. Shiga, R. Iizuka, S. Ueda, K. Demachi, D. Yamafuji, H. Kayama, T.-a. Yamagishi, *J. Am. Chem. Soc.* **2012**, *134*, 20322-20325.

[4] J. Zhang, X. Bao, J. Zhou, F. Peng, H. Ren, X. Dong, W. Zhao, *Biosens. Bioelectron.* **2016**, *85*, 164-170.

[5] T. Ogoshi, R. Shiga, T.-a. Yamagishi, *J. Am. Chem. Soc.* **2012**, *134*, 4577-4580.

[6] H. Zhang, X. Ma, K. T. Nguyen, Y. Zhao, *ACS Nano* **2013**, *7*, 7853-7863.
